# Recent advances in Cu-catalyzed C(sp^3^)–Si and C(sp^3^)–B bond formation

**DOI:** 10.3762/bjoc.16.67

**Published:** 2020-04-15

**Authors:** Balaram S Takale, Ruchita R Thakore, Elham Etemadi-Davan, Bruce H Lipshutz

**Affiliations:** 1 Department of Chemistry & Biochemistry, University of California, Santa Barbara, California 93106, USA

**Keywords:** C–B bonds, copper catalysis, C–Si bonds, enantioselective reactions, sp^3^ carbon functionalization

## Abstract

Numerous reactions generating C–Si and C–B bonds are in focus owing to the importance of incorporating silicon or boron into new or existing drugs, in addition to their use as building blocks in cross-coupling reactions en route to various targets of both natural and unnatural origins. In this review, recent protocols relying on copper-catalyzed sp^3^ carbon–silicon and carbon–boron bond-forming reactions are discussed.

## Introduction

**Scheme 1:**
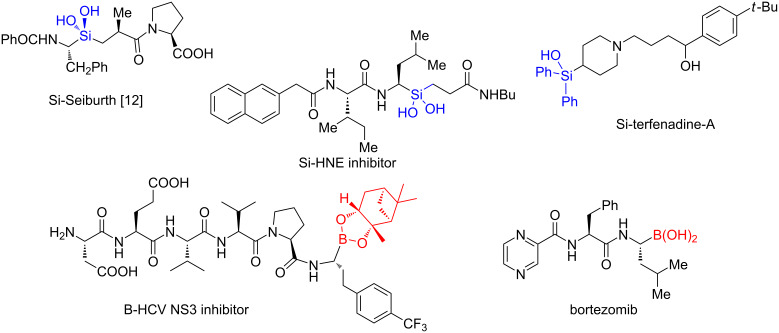
Pharmaceuticals possessing a silicon or boron atom.

Transition-metal-catalyzed silylation and borylation are useful transformations [1], widely studied because organosilicon [2–3] and organoboron compounds [4] are common partners in a variety of important cross-coupling reactions. They are also especially valuable precursors to other functional groups such as organic halides, alcohols [5–7], etc. Moreover, recent drug discovery efforts have shifted towards the incorporation of these non-natural functional groups into existing or new drugs. Examples include the incorporation of silicon bioisosteres that help increase lipophilicity, subsequently altering the existing metabolic pathway of a drug due to differences in its physicochemical properties [8]. On the other hand, the trigonal planar nature of boron can lead to dative bond formation with enzymes, and therefore increase binding affinity. As shown in Scheme 1, several silicon [9–12] and boron-containing [13–16] drugs have already entered the market, or are currently in the drug development pipeline. As the number of drugs containing these functional groups continues to increase, new synthetic pathways for their inclusion will surely attract synthetic organic chemists, as well challenge them to consider both existing approaches and developing new reactions.

Many recent reviews already cover the syntheses and applications of organosilicon [17–20] and organoboron [21–24] compounds (i.e., C(sp^3^)–Si and C(sp^3^)–B). Nonetheless, considering the extent of their use and their increasing popularity in the pharmaceutical industry, as well as the significant growth in the development of Cu-catalyzed processes applied to their syntheses, a review on this subject seems quite timely. Therefore, we will focus on highlights of the past 5–6 years in this area, dividing the document into two sections: C–Si and C–B bond formation.

## Review

### Cu-catalyzed C–Si bond formation

#### 1.1 Substitution reactions

**Scheme 2:**
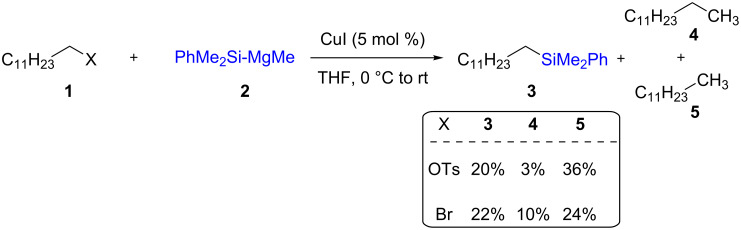
The first Cu-catalyzed C(sp^3^)–Si bond formation.

Alkylsilanes are an interesting class of substrates for medicinal and polymer chemistry. Nevertheless, they can be transformed into a variety of building blocks for subsequent use in complex molecule synthesis. The first example of a copper-catalyzed C(sp^3^)–Si bond formation was reported by Oshima and co-workers in 1984 [25]. In an attempt to determine the existence of radical behavior of PhMe_2_Si-MgMe (**2**), they studied the reaction of this Grignard reagent with dodecyl tosylate (**1**, X = OTs), which led to the formation of dodecyl silane **3** (20%) along with tridecane **4** (3%) and dodecane **5** (36%). Similarly, dodecyl bromide (**1**, X = Br) led to the same three products in 22%, 10%, and 24% yields, respectively (Scheme 2).

**Scheme 3:**
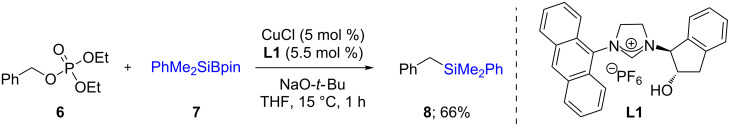
Conversion of benzylic phosphate **6** to the corresponding silane.

**Scheme 4:**
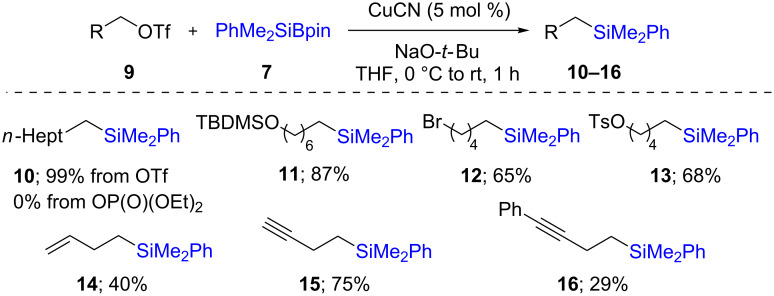
Conversion of alkyl triflates to alkylsilanes.

Later, a far higher yielding C–Si bond formation (66%) was developed by Hayashi [26]. More precisely, they used a catalytic amount of the complex formed from CuCl and NHC ligand **L1**, together with Suginome’s reagent (**7**), to successfully convert benzyl phosphate **6** to benzylic silanes **8**. Curiously, the reaction proceeded even in the absence of a ligand, albeit with lower yield (25%; Scheme 3). However, only one example was reported and a more general method for the preparation of alkylsilanes was developed by the Oestreich group in 2016 [27]. The reaction could be performed using CuCN as catalyst in the absence of a ligand. A wide variety of triflates **9**, including some containing a remote tosylate, bromide, alkene, or alkyne functionality, afforded the desired alkylsilanes **10**–**16** in fair to good yields (Scheme 4). It should be mentioned that secondary triflates or halides are not suitable for this transformation, since they lead to products resulting from fast β-elimination.

**Scheme 5:**
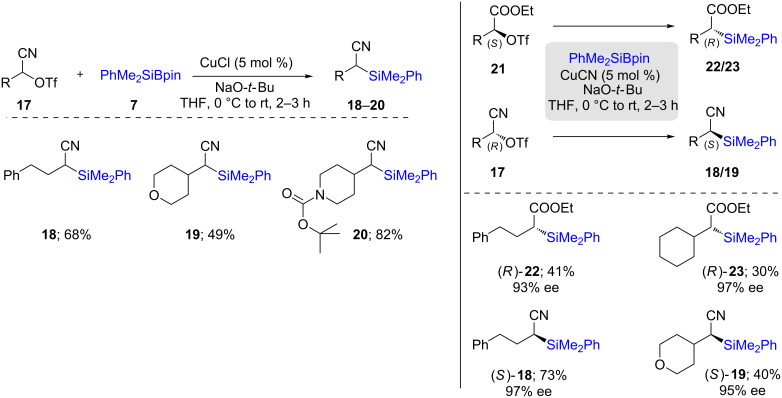
Conversion of secondary alkyl triflates to alkylsilanes.

Considering this limitation, they designed alkyl triflates stabilized by a strong electron-withdrawing group (EWG) directly attached to the triflate bearing carbon. In this way, different classes of substrates bearing either a nitrile (**17**) or ethyl ester (**21**) easily underwent conversion to the corresponding silylated products **18**–**20** in moderate to good yields (Scheme 5). This method was extended to the use of enantiomerically pure substrates, and as expected, inversion of configuration occurred at the stereogenic carbon centers, where an *S*-configured substrate led to an (*R*)-product (**22/23**)*,* and vice versa (**18/19**) [28].

**Scheme 6:**
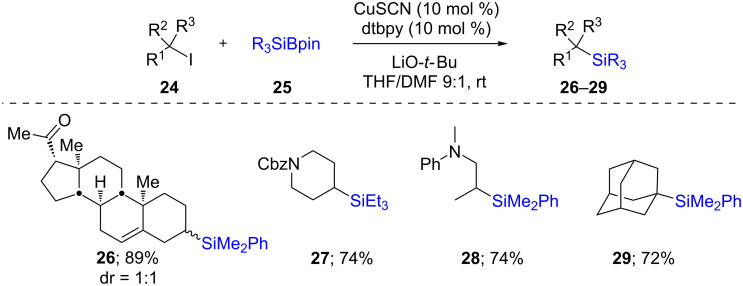
Conversion of alkyl iodides to alkylsilanes.

**Scheme 7:**
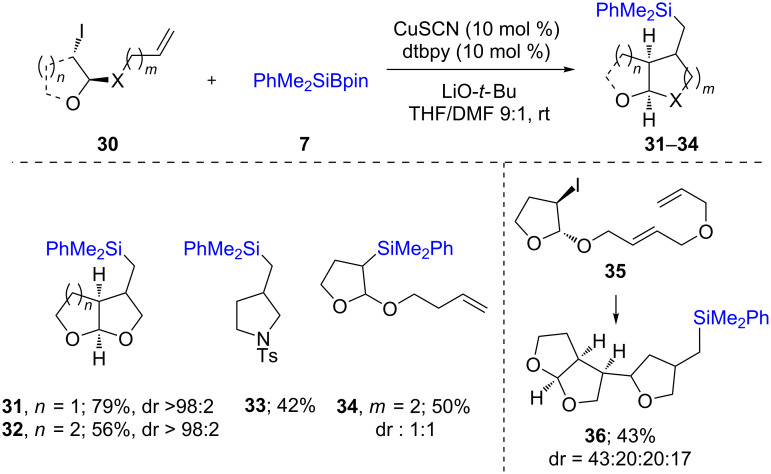
Trapping of intermediate radical through cascade reaction.

**Scheme 8:**
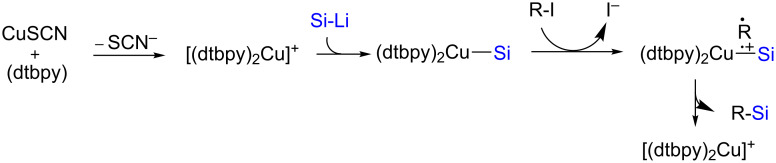
Radical pathway for conversion of alkyl iodides to alkylsilanes.

While the mechanism for the above two protocols are essentially ionic, an alternative method was reported by Oestreich [29] for the synthesis of secondary (**26**–**28)** and tertiary (**29**) silanes via a radical pathway, in good chemical yields (Scheme 6). It was possible to perform this reaction on a wide array of alkyl iodides using various silylating reagents. One selected application of this reaction, which also supports a radical mechanism, is depicted in Scheme 7. Here, an intermediate radical could easily be intercepted by a tethered alkene in **30** leading to a 5-*exo-trig* cyclization and hence, the formation of a 5-membered ring as found in products **31**–**33**. The formation of, e.g., compound **34**, suggested that a specific geometry of the tethered alkene is required, as no 6-membered ring formation was observed. Interestingly, Cárdenas’ precursor **35** [30] led, after a cascade of reactions, to the formation of compound **36**. Computational as well as experimental studies suggested that the ligand and thiocyanate anion both play specific roles in the generation of an active Cu species able to trap the Si–Li (previously formed by reaction of LiO-*t-*Bu(THF)_3_ with PhMe_2_Si-Bpin; other atoms on Si and Li were omitted for clarity). The resultant Cu–Si species may then form a dative bond with the alkyl iodide leading to an electron transfer to the iodine atom, thereby liberating iodide, an alkyl radical, and a radical cation of the Cu complex. Recombination of the latter radicals leads to the formation of the desired silane along with the regeneration of the active Cu species (Scheme 8).

**Scheme 9:**
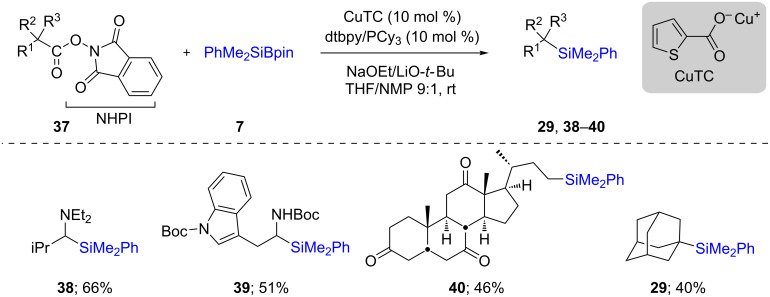
Conversion of alkyl ester of *N*-hydroxyphthalimide to alkylsilanes.

This strategy was also explored on redox-active alkyl esters derived from *N*-hydroxyphthalimide (NHPI, **37**), in which case the reactions proceeded through a similar radical pathway due to, in part, the alkyl radical surrogate nature of the NHPI esters. The radical generated via decarboxylation of these esters is easily trapped by the in situ-formed Cu–Si species leading ultimately to the formation of the desired C–Si bond. This process could be applied to a variety of substrates leading to the desired products **29** and **38**–**40** (Scheme 9) [31].

**Scheme 10:**
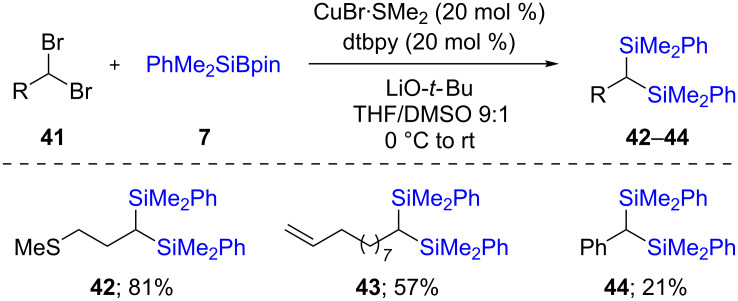
Conversion of *gem*-dibromides to bis-silylalkanes.

This silylation reaction was also performed on geminal dibromides **41**. In such cases, both bromides were replaced by silyl groups. The reaction supposedly occurred partly via ionic silylation (of the first bromide), and partly via a radical pathway (silylation of the second bromide), effected by the presence of catalytic amounts of CuBr·SMe_2_ along with dtbpy as ligand. Whatever the mechanism(s), a variety of substrates were suitable for this transformation, giving the desired products **42**–**44** in good chemical yields (Scheme 10) [32].

#### 1.2 Additions to imines

**Scheme 11:**
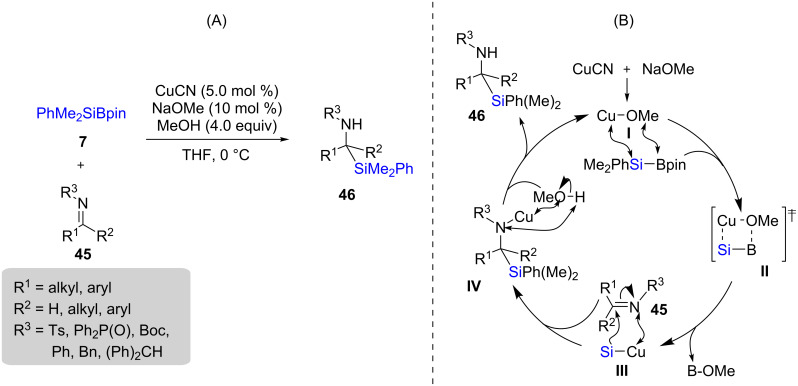
Conversion of imines to α-silylated amines (A) and the reaction pathway (B).

Among the very first studies on Cu-catalyzed additions to imines one can include the work of Moeller and co-workers published in 2002 in which they performed 1,2-additions of a silyl moiety to iminium ions [33], based on earlier work using silyllithium intermediates [34]. More recently, the Oestreich group described a more general method for the addition of silyl moieties to aldimines or ketimines **45** using 5 mol % CuCN together with Suginome’s PhMe_2_Si-BPin [35] as a silicon pro-nucleophile (Scheme 11A) [36]. The mechanism followed the expected pathway involving transmetallation from boron to copper to form the corresponding Cu–Si species **III**. This intermediate then adds to the imine **45** to give intermediate **IV**, which after undergoing proto-demetallation afforded the final product **46** (Scheme 11B).

**Scheme 12:**
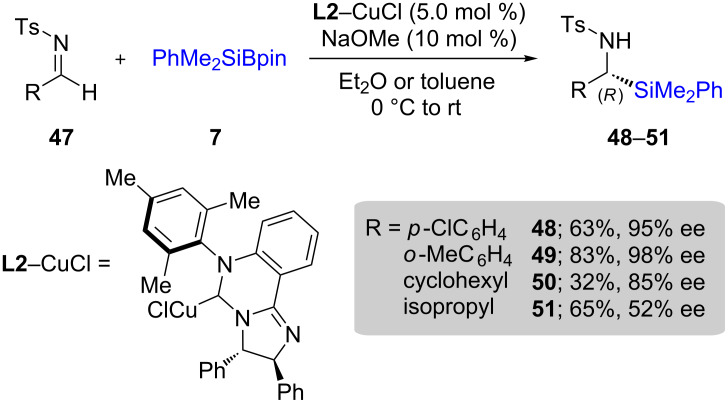
Conversion of *N*-tosylimines to α-silylated amines.

Initially, the reaction was limited to the generation of racemic products. Ultimately, optimization using McQuade’s six membered *N*-heterocyclic carbene (NHC) **L2** [37] in combination with CuCl led to conditions applicable to different types of aldimines **47** to give both good chemical yields and enantioselectivities associated with products **48**–**51** (Scheme 12). However, these additions were not applicable to neutral imines or ketimines – they are best performed on activated imines (e.g., *N*-sulfonyl imines) [38].

**Scheme 13:**
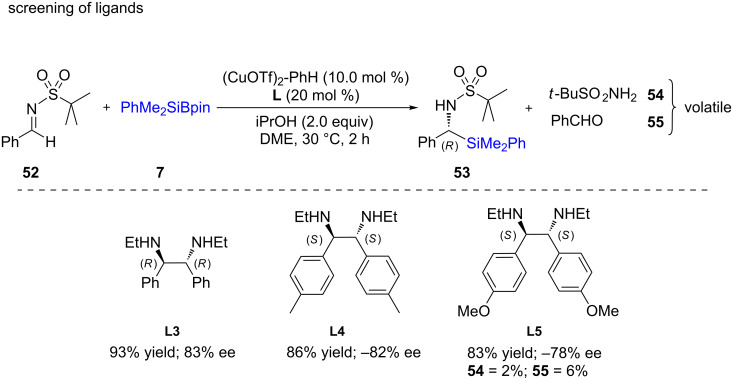
Screening of diamine ligands.

**Scheme 14:**
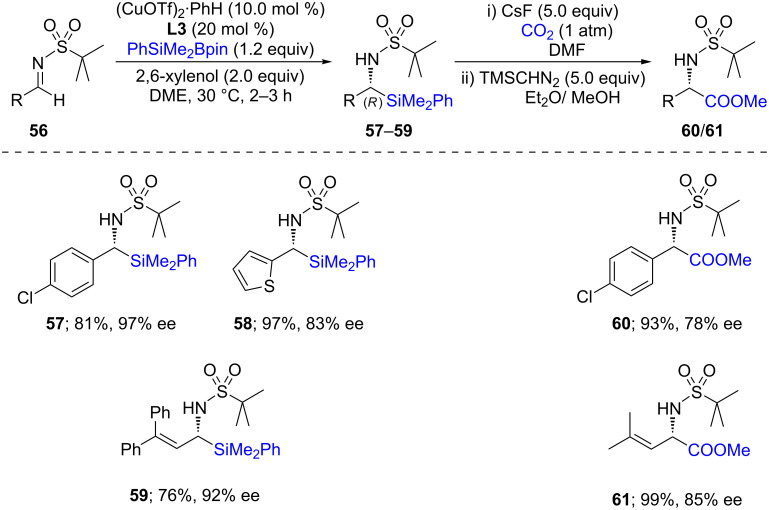
Conversion of *N*-*tert*-butylsulfonylimines to α-silylated amines.

The same year, Sato and co-workers [39] used a comparatively simple ligand to perform asymmetric silylations of imines. Different ethylenediamine-based ligands (**L3**–**L5)** were screened from which **L3** proved to give even better yields and ees (Scheme 13). The use of this ligand was explored with other substrates **56** and found to give excellent chemical yields with good enantioselectivities for products **57**–**59** (Scheme 14). Interestingly, this approach could be extended to the synthesis of amino acids using cesium fluoride in the presence of CO_2_ gas to afford **60** and **61**. From the representative examples shown below, it appears that α-amino esters could be obtained in excellent chemical yields with little erosion in enantiopurity.

**Scheme 15:**
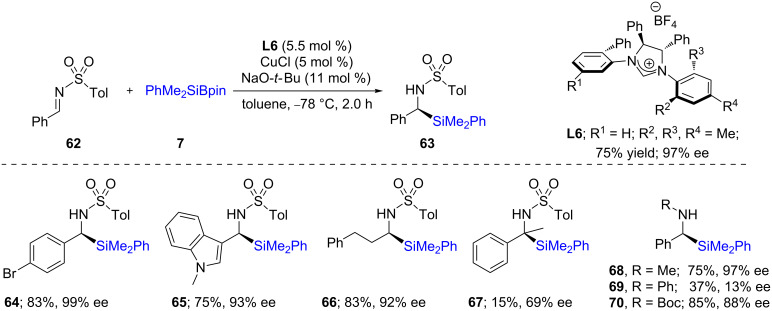
Conversion of aldimines to nonracemic α-silylated amines.

Similar work was described by Zhao et al. using a C1-symmetric chiral NHC ligand **L6** together with catalytic amounts of CuCl. Here again, reactions could be performed on numerous albeit activated aldimines **62**, failing to use unactivated educts [40]. Likewise, the α-silylated sulfonamide **67** was obtained in modest ee, although the chemical yield of the transformation was very low (15%). Changing the protecting group from Ts to methyl (**68**) or Boc (**70**) in the aldimine series did not alter yields or ees significantly. However, with the aniline derivative **69** (R = Ph on nitrogen), the ee dropped precipitously (Scheme 15).

**Scheme 16:**
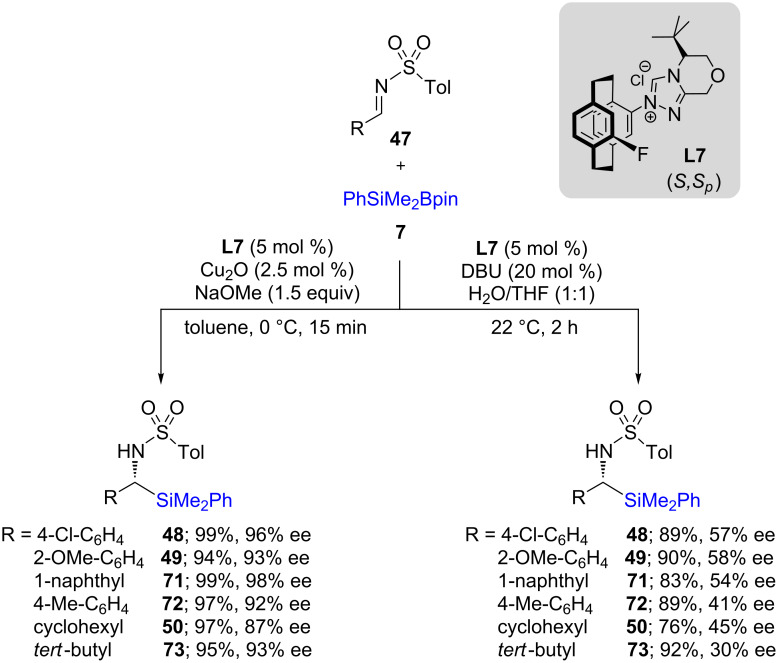
Conversion of *N*-tosylimines to α-silylated amines.

Chen et al. used a paracyclophane-based NHC ligand, along with Cu_2_O, to perform similar asymmetric 1,2-silyl additions onto activated imines. For several substrates (**47**) studied, the chemical yields were higher than in previously reported reactions, along with higher ees (Scheme 16, left). Although the synthesis of the ligand was somewhat tedious, the reaction proceeded in shorter reaction times [41]. Another approach, also shown below (Scheme 16, right), involves a metal-free background reaction. This represents the first example done in a water/THF solvent system at room temperature, giving moderate chemical yields and ees. It has been proposed that the ligand itself acts as an organocatalyst, eliminating the need for a copper catalyst.

#### 1.3 Additions to aldehydes

**Scheme 17:**
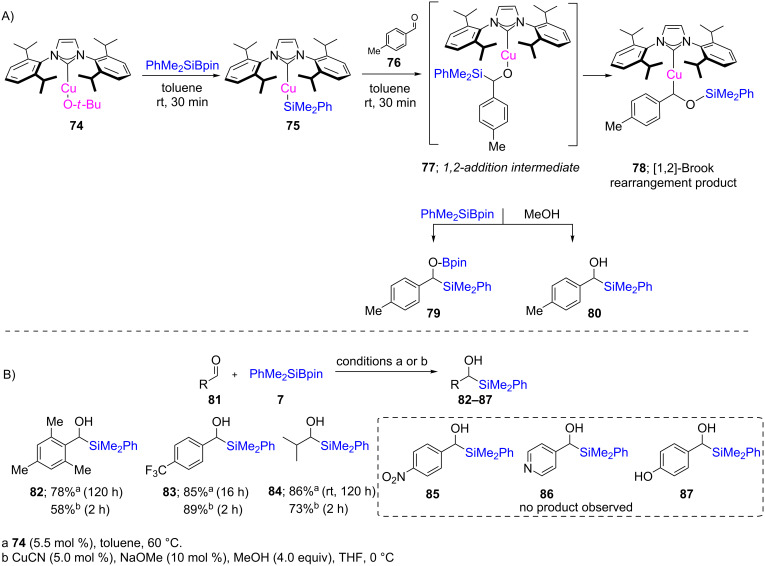
Reaction pathway [A] and conversion of aldehydes to α-silylated alcohols [B].

The Oestreich group [42] described NHC–Cu (**74**)/base 1,2-additions at room temperature of silicon pro-nucleophiles onto aldehydes **81** leading to racemic silyl alcohols **82**–**87** (Scheme 17B). A detailed mechanistic study revealed that a Cu–Si species is involved, giving intermediate **77**, which subsequently undergoes fast Brook rearrangement to form a C–Cu bond-containing intermediate **78** (Scheme 17A). The O–Cu intermediate **77** can be intercepted either by PhMe_2_SiBpin or methanol to give the silylated O-Bpin product **79** or silyl alcohol **80**, respectively. Interestingly, it was eventually realized that the use of the NHC ligand is not required if CuCN is used as the catalyst. Under such conditions, the desired product could be obtained faster than when the NHC was used. However, aldehydes containing reactive functional groups, as in **85**–**87**, gave none of the desired product with either CuCN or the Cu–NHC complex **74**.

**Scheme 18:**
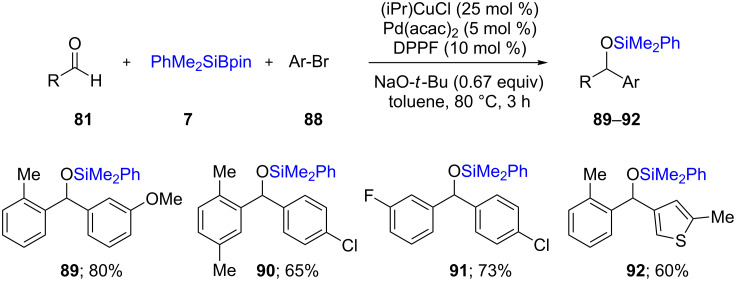
Conversion of aldehydes to benzhydryl silyl ethers.

On the other hand, Ohmiya and co-workers envisioned inverting the polarity of an aldehyde **81** via conversion into the corresponding α-alkoxyalkyl Cu(I) anion **78**. Utilizing **78**, which undergoes transmetallation to an initially formed Pd(II) intermediate (from oxidative addition) led to cross couplings affording benzhydryl silyl ethers **89**–**92** (Scheme 18), thus showcasing the synergistic relationship between Pd and Cu catalysis [43].

**Scheme 19:**
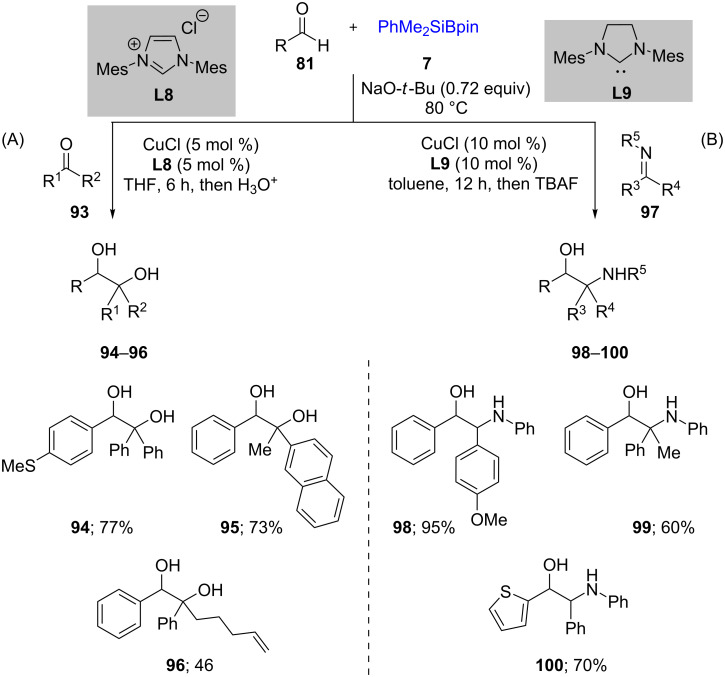
Conversion of ketones to 1,2-diols (A) and conversion of imines to 1,2-amino alcohols (B).

Driven by the success of earlier results, the authors utilized **78** for reductive couplings between ketones **93** and imines **97** as electrophiles to form unsymmetrical 1,2-diols **94**–**96** and 1,2-amino alcohols **98**–**100**, respectively. Palladium was no longer needed for these transformations. The scope of the reaction with ketones was not limited to diaryl species and aryl ketones participated in the reaction, including those with more hindered alkyl groups (Scheme 19) [44].

**Scheme 20:**
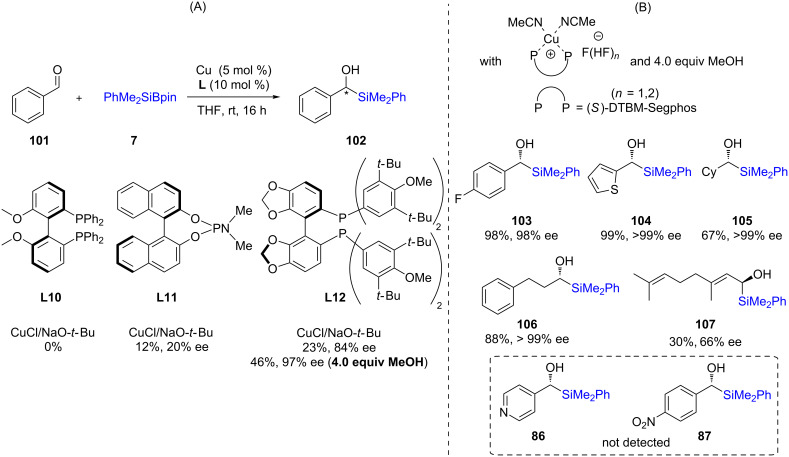
Ligand screening (A) and conversion of aldehydes to α-silylated alcohols (B).

**Scheme 21:**
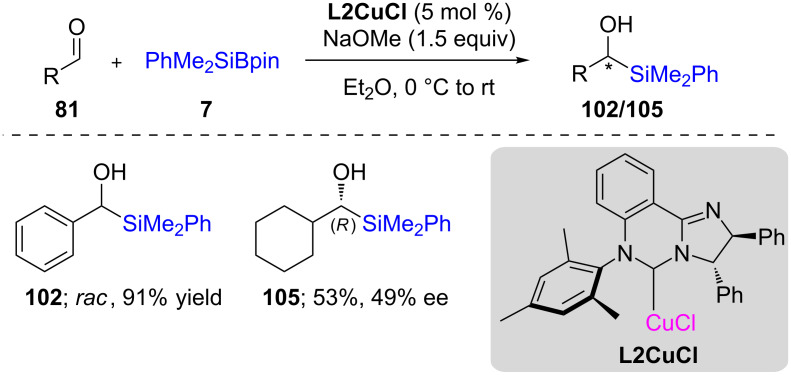
Conversion of aldehydes to α-silylated alcohols.

This discovery was followed by a study from the Riant group [45] that performed asymmetric 1,2-additions on aldehydes using Suginome’s reagent in the presence of phosphine-ligated copper (Scheme 20A). After optimization, they found that DTBM-Segphos (**L12**; 10 mol %) together with CuCl (5 mol %) led to moderate yields (46%) and high ee (97%) for this transformation. In an attempt to improve the chemical yield, they prepared a preformed complex of diphosphine copper(I) difluoride, which, indeed, increased the yield to 87% together with raising the ee to 99%. A variety of substrates were then studied using this complex, and in each case, more than 90% ee along with high chemical yields were obtained (Scheme 20B). Simultaneous efforts by Oestreich were also directed towards nonracemic silyl alcohol synthesis using McQuade’s NHC **L2** (Scheme 21) [46].

#### 1.4 Additions to unsaturated compounds

**Scheme 22:**
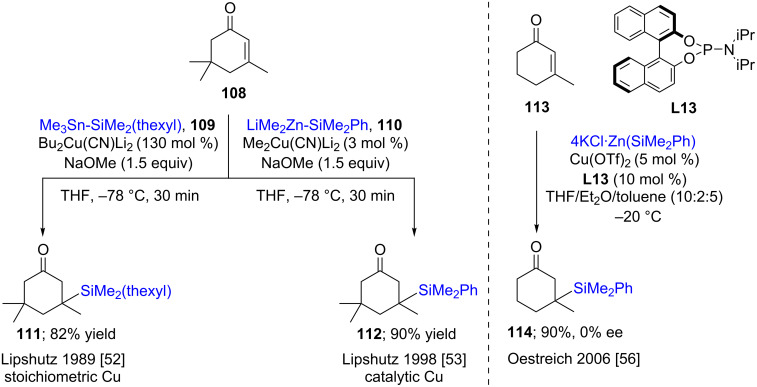
1,4-Additions to α,β-unsaturated ketones.

Back in 1977, Fleming first described 1,4-additions of cuprates derived from a silyllithium [47] to α,β-unsaturated ketones [48]. There was no effort made at that time to convert these reactions to the corresponding catalytic processes, rather, the accent was more towards using the silyl group introduced as a hydroxy group equivalent [49–51]. Similarly, although the Lipshutz group [52] also showed that in situ-formed trialkylsilylcuprates could be used for 1,4-additions with unsaturated ketones **108** in high chemical yields (Scheme 22, left), here, too, a catalytic method had yet to be reported limiting the use of these methods. Almost a decade later, however, Lipshutz et al. revisited this reaction, showing that it could be performed using only 3 mol % of the copper catalyst, while leading to high chemical yields of the desired silylated product **112** (Scheme 22, left) [53]. In the same year, Hosomi and co-workers reported that the alternative silane, 1,1,2,2-tetramethyl-1,2-diphenyldisilane, also commercially available, can be used for nucleophilic additions to α,β-unsaturated ketones. Thus, by cleaving the Si–Si bond in the presence of a Cu(I) salt, an active [Cu–Si] species is generated leading to β-silylated ketones [54]. More than a decade later, Molander et al. made use of the same disilane as a source of the nucleophile for additions to α,β-unsaturated alkenes and alkynes as Michael acceptors bearing sulfones, nitriles, cyano, amido, and carboxyl ester groups to form β-silylated alkanes and alkenes in good to moderate yields [55]. Following this report, Oestreich and co-workers examined asymmetric additions of silicon to unsaturated ketones **113** using P–N-type ligand **L13**. However, the background reaction of the silyl–zinc reagent was predominant leading to poor chirality transfer from the phosphine ligand **L13**, giving essentially the racemic product **114** (Scheme 22, right) [56].

**Scheme 23:**
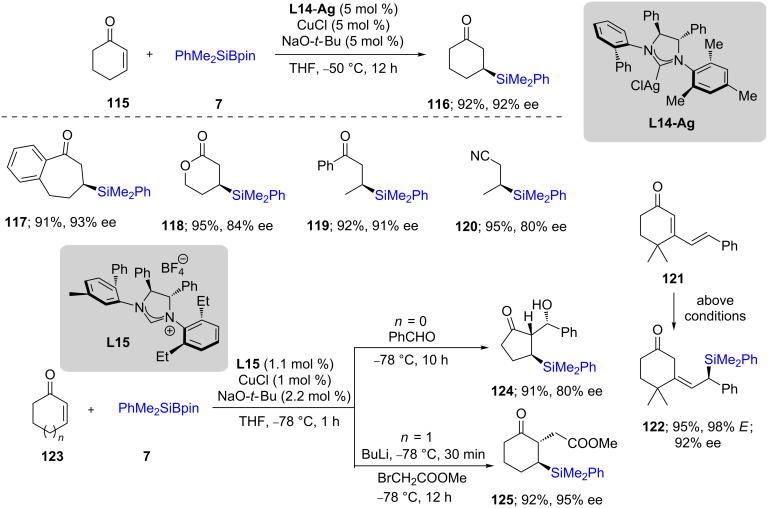
1,4-Additions to unsaturated ketones to give β-silylated derivatives.

In 2010, Hoveyda [57] used CuCl along with an NHC ligand (**L14**) that enabled 1,4-additions of silicon nucleophiles to unsaturated ketones. The best results were obtained at low temperatures, giving both high chemical yields and products with high enantiomeric ratios. Both cyclic (**117** and **118**) and acyclic (**119**,**120**) ketones could be silylated efficiently (Scheme 23). Interestingly, cyclic enones conjugated to an external double bond, as in **121**, upon exposure to these conditions resulted in an excellent selectivity for the 1,6-addition and in high ee for product **122**. Moreover, the intermediates could be trapped in the presence of electrophiles, such as aldehydes or alkyl halides to afford interesting α-substituted products **124** and **125**. This phenomenon was further studied in detail on different dienones [58].

**Scheme 24:**
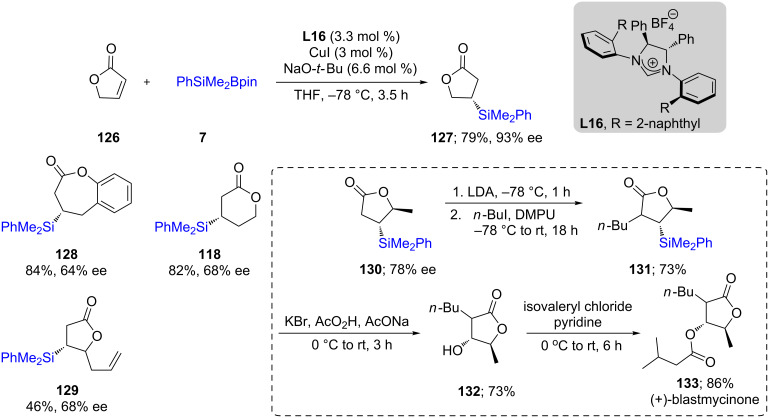
Additions onto α,β-unsaturated lactones to give β-silylated lactones.

In 2013, Procter and co-workers [59] extended the scope of this silicon addition to unsaturated lactones using copper catalysis together with NHC ligand **L16**. The ligand used previously to great advantage by the Hoveyda and Oestreich groups for conjugate additions did not yield high ees for these substrates. The authors designed a new NHC possessing either an anthryl or naphthyl group, the use of which led to moderate to good enantioselectivities in most cases. An application of this method is shown for the total synthesis of the natural product (+)-blastmycinone (Scheme 24).

**Scheme 25:**
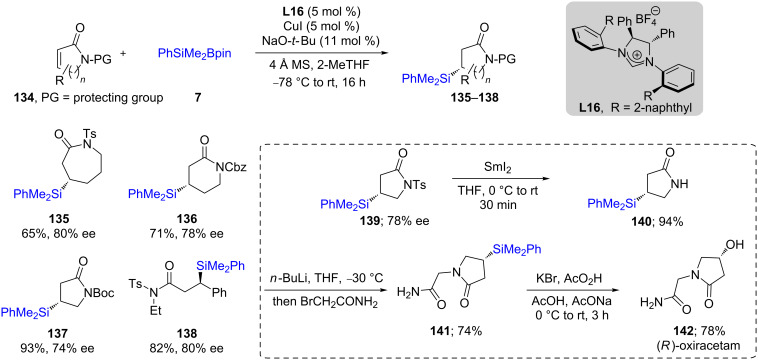
Conversion of α,β-unsaturated to β-silylated lactams.

As a follow up to this work, the same authors successfully accomplished, for the first time, a silyl transfer to lactams in both high chemical yields and high ees [60]. In this case, a comparatively higher (5 mol %) copper loading was necessary. Not only lactams (Scheme 25) but also acyclic, unsaturated amides could be efficiently silylated under these conditions. Interestingly, this protocol was applied to the total synthesis of (*R*)-oxiracetam (**142**), a drug used for the treatment of Alzheimer’s disease [61].

**Scheme 26:**
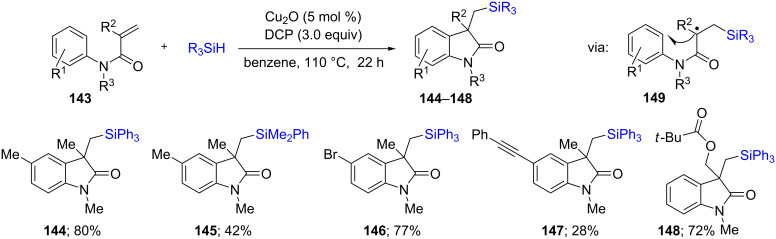
Conversion of *N*-arylacrylamides to silylated oxindoles.

In a similar way, a ligand-free, intramolecular silylarylation of unsaturated amides **143** could be performed, albeit following a radical pathway leading to cyclic products (Scheme 26). From the different radical generators screened, dicumyl peroxide (DCP) was found to be very effective in leading to the corresponding products **144**–**148**. Along with different acrylamides, different hydrosilanes were used as silyl source and the reaction could be applied to a wide variety of substrates, although the methodology required hazardous conditions; i.e., hot benzene [62].

**Scheme 27:**
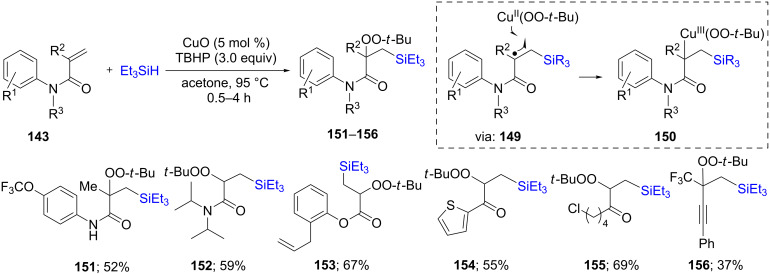
Conversion of α,β-unsaturated carbonyl compounds to silylated *tert-*butylperoxides.

A few years later, similar types of reactions were carried out by Loh and co-workers [63]. In this case, a variety of α,β-unsaturated compounds, that, rather than undergoing intramolecular cyclization, could be intercepted at the intermediate radical stage (**149**) with radical initiator TBHP present in excess, leading to silylated peroxy products **151**–**156**. This approach was applied to different types of conjugated systems, including esters, ketones, amides, alkynes, etc. (Scheme 27).

**Scheme 28:**
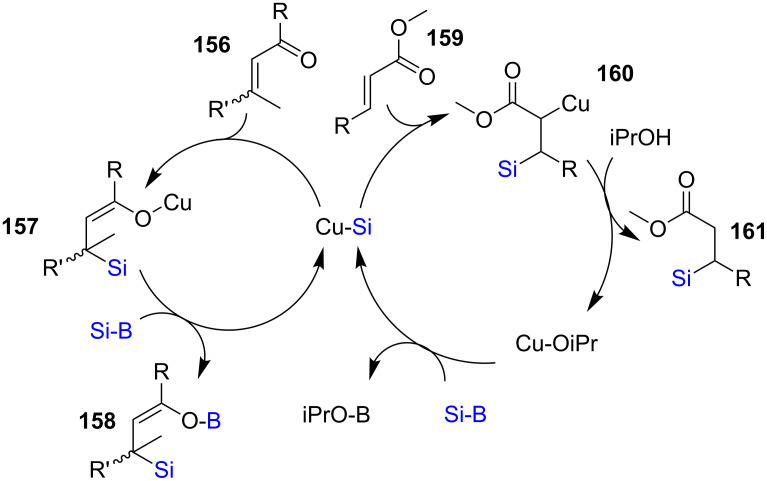
Catalytic cycle for Cu(I) catalyzed α,β-unsaturated compounds.

**Scheme 29:**
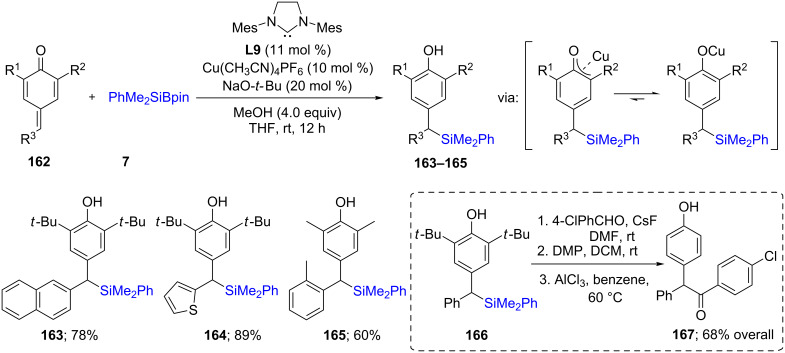
Conversion of *p*-quinone methides to benzylic silanes.

Kleeberg [64] et al*.* have contributed to the understanding of the mechanism of silylation reactions of unsaturated compounds. In their studies, it was possible, surprisingly, to isolate and characterize a β-silyl boron enolate complex **158**. On the basis of experimental and NMR studies, they proposed that for α,β-unsaturated ketones, a 1,4-addition product was formed through a transient copper enolate, **157**. With α,β-unsaturated esters, however, a carbon enolate, **160**, is the intermediate. In order to maintain a catalytic cycle, iPrOH could be added to regenerate the active Cu species (Scheme 28). This study again proofed that an enol system, as in the case of unsaturated ketones which is not easy to isolate, could be modified in such way that the resultant intermediate is highly stable. For example, Tortosa [65] showed that this type of catalytic system could be applied to the quinone methide system **162**, which is equivalent to dienones (Scheme 29). In this case, however, instead of forming an enol, a highly stable phenol resulted from the addition of silicon at the methide position. The reaction was exclusively done on electron-rich systems **163**–**166**. Nonetheless, further functionalization of one of the silicon-containing products (**166**) was carried out to arrive at the keto phenol derivative **167**.

**Scheme 30:**
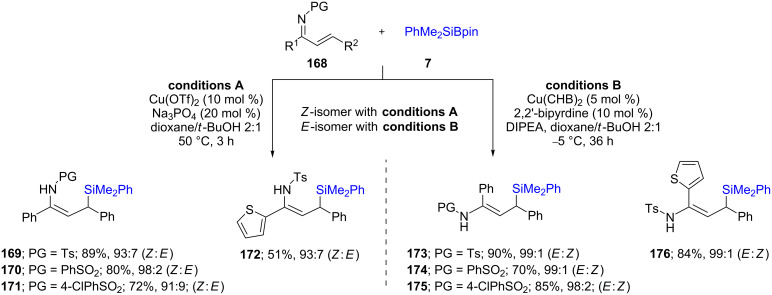
Conversion of α,β-unsaturated ketimines to regio- and stereocontrolled allylic silanes.

**Scheme 31:**
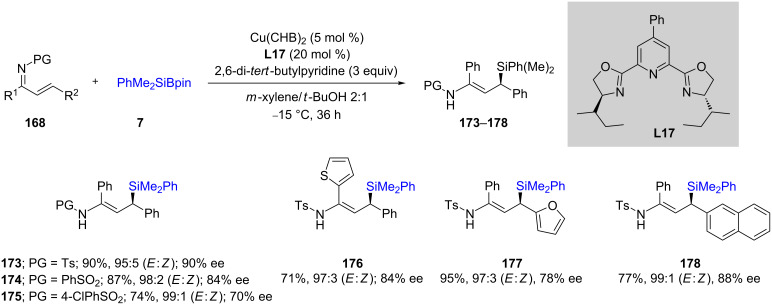
Conversion of α,β-unsaturated ketimines to enantioenriched allylic silanes.

Loh, Xu, and co-workers [66] explored related reactions involving unsaturated ketimines using copper triflate or copper bis(4-cyclohexylbutyrate) (Cu(CHB)_2_) to prepare allylic silanes. Interestingly, using one or the other of these two copper sources and a selection of appropriate reaction conditions allowed for the complete reversal in geometrical isomers of the products. For example, the use of Cu(OTf)_2_/Na_3_PO_4_ in dioxane/*t*-BuOH resulted in *Z*-isomer formation (93% selectivity; Scheme 30, left; conditions A), while the use of catalytic Cu(CHB)_2_/diisopropyl ethylamine in *m*-xylenes/*t*-BuOH gave the *E*-isomer (99% selectivity; Scheme 30, right; conditions B). They also screened various bisoxazoline-containing ligands (for example Scheme 31, **L17**) for chiral induction which led to good enantioselectivities, in addition to excellent *E:Z* ratios within the resulting alkenes. This methodology could be applied to a wide variety of compounds, including those containing heterocycles (**176**–**178**).

**Scheme 32:**
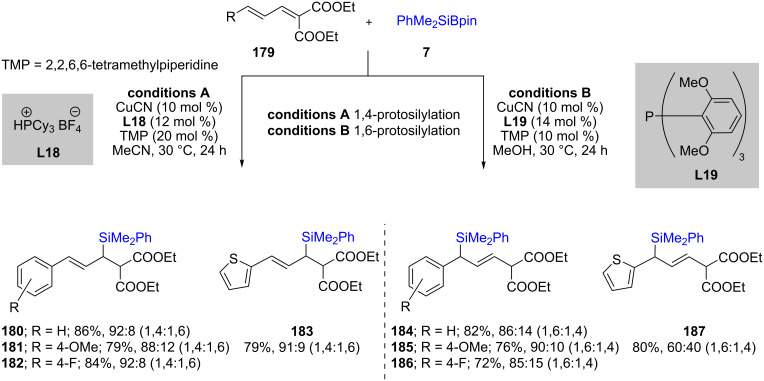
Regioselective conversion of dienedioates to allylic silanes.

In a later study [67], they found that by changing ligands, a dramatic switch in the selectivity could be induced in silyl additions to dienedioates. Interestingly, tricyclohexylphosphonium tetrafluoroborate (**L18**), along with CuCN, resulted in the 1,4-addition as the major pathway (Scheme 32, left). By contrast, the use of the bulkier tris(2,6-dimethoxyphenyl)phosphane (**L19**) led to the formation of 1,6-adducts (Scheme 32, right).

**Scheme 33:**
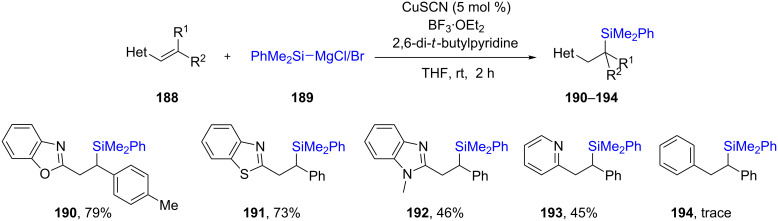
Conversion of alkenyl-substituted azaarenes to β-silylated adducts.

**Scheme 34:**
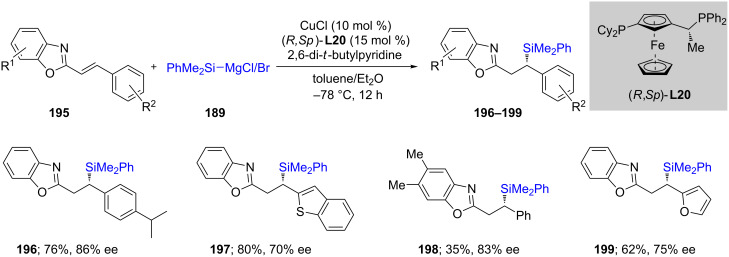
Conversion of conjugated benzoxazoles to enantioenriched β-silylated adducts.

The Oestreich group [68] used silicon-based Grignard reagents **189** to add to conjugated heteroaromatics **188**, e.g., benzoxazole (as an extension to more commonly studies ketones, esters, imines, etc.), leading to products **190**–**194**. The heterocycle played a crucial role, as in its absence, none of the expected product was obtained, e.g., when simple stilbene was used the reaction led to only traces of product **194** (Scheme 33). With benzoxazole-conjugated alkenes, upon treatment with catalytic Cu complexed by a nonracemic Josiphos ligand (**L20**), good chemical yields of the desired enantioenriched products **196**–**199** could be isolated (Scheme 34).

**Scheme 35:**
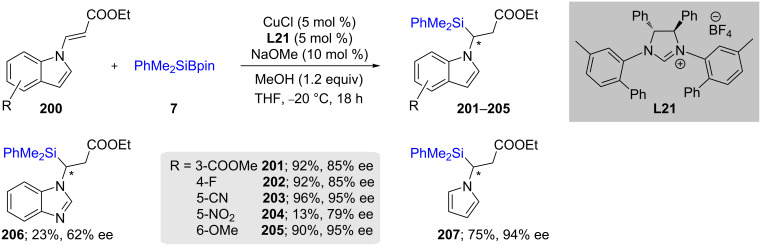
Conversion of α,β-unsaturated carbonyl indoles to α-silylated *N*-alkylated indoles.

Another class of heterocycles, α-silylated *N*-alkylated indoles **201**–**205** recently reported by Xu and co-workers, were formed using a nonracemic Cu–NHC catalyst through the enantioselective addition of the PhMe_2_Si group to α,β-unsaturated carbonyl indoles **200** (Scheme 35). The authors demonstrated a wide substrate scope, including an example of benzimidazole and pyrrole, although with varying chemical yields [69].

**Scheme 36:**
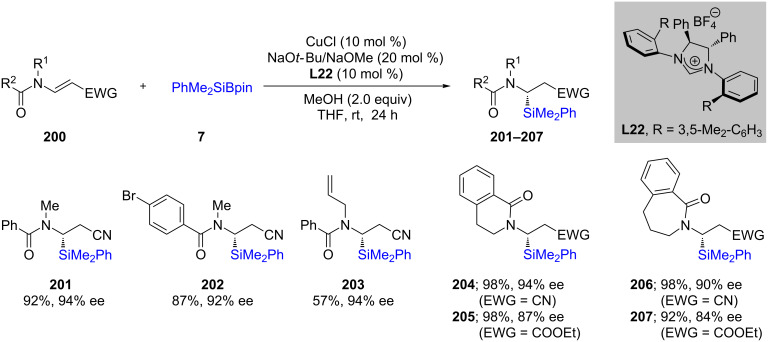
Conversion of β-amidoacrylates to α-aminosilanes.

Recently Zhang et al. [70] explored various **L22**-containing (NHC)Cu catalysts by means of generating interesting nonracemic aminosilanes. In this case, they performed 1,2-additions on β-amidoacrylates (**200**) using 10 mol % CuCl in THF at room temperature (Scheme 36). In most cases, the chemical yields were very good to excellent, as were the resulting ees of the products **201**–**207**.

**Scheme 37:**
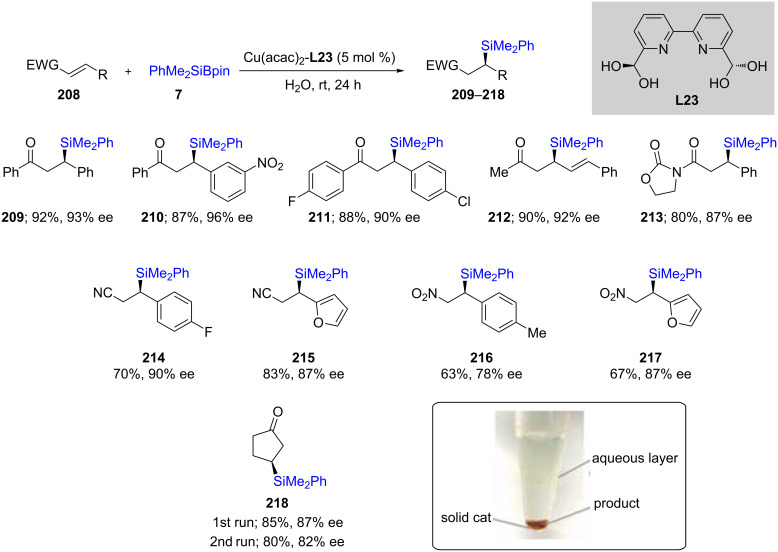
Conversion of α,β-unsaturated ketones to enantioenriched β-silylated ketones, nitriles, and nitro derivatives in water.

The processes described above were run using stoichiometric copper and organic solvents at cryogenic temperatures, hence, they are neither economical nor sustainable [71–73]. Alternatively, the Kobayashi [74] group has shown that such reactions can be done not only more efficiently, but in a far “greener” fashion using water as the solvent, catalytic amounts of copper, and the reaction being done at room temperature. One of the biggest advantages of using water was that this catalytic system behaved as if being run homogeneously in an organic solvent. However, due to a lack of solubility of both the substrate and catalyst in water, it is actually heterogeneous and hence, provided an opportunity for recycling and reuse. This catalyst, therefore, was isolated using simple centrifugation and reused in a second reaction leading to no appreciable loss in catalytic activity en route to product **218**. It was also utilized not only for unsaturated ketones but also to deliver the PhMe_2_Si moiety in a 1,4-manner to unsaturated nitro and cyano derivatives leading to adducts **209**–**218**, each being obtained in high chemical yield and good enantioselectivity (Scheme 37).

#### 1.5 Miscellaneous reactions

**Scheme 38:**
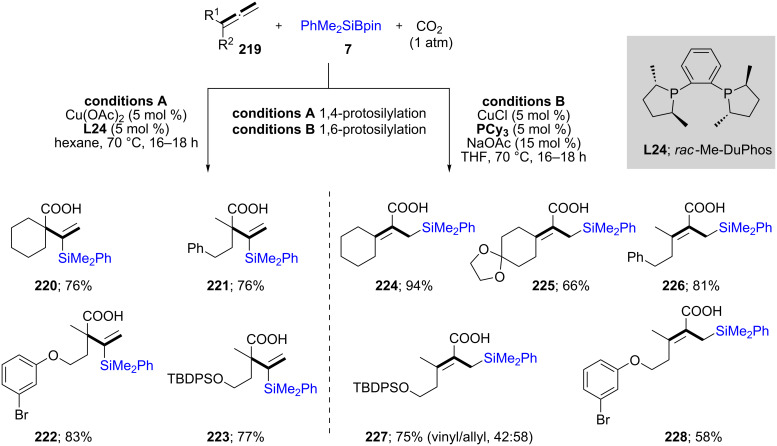
Regio-divergent silacarboxylation of allenes.

The Tsuji group developed a mild method for the regio-divergent silacarboxylation of allenes **219**. Based on the type of ligands used (e.g., Me-DuPhos; **L24** vs. Cy_3_P), either vinyl (**220**–**223**) or allylic (**224**–**228**) silanes could be obtained, respectively, in good yields. Different types of substrates were studied to give maximum selectivity for the desired product (Scheme 38). Also, for cases forming allylic silanes, only *Z*-isomers were obtained [75].

**Scheme 39:**
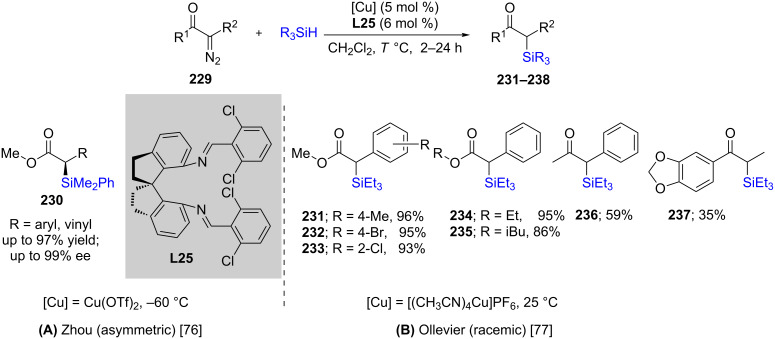
Silylation of diazocarbonyl compounds, (A) asymmetric and (B) racemic.

In furthering Zhou’s initial report [76], Ollevier and co-workers [77] recently described carbene insertions, starting with **229**, into Si–H bonds, leading to a wide variety of silylated products [18]. The original work by Zhou included asymmetric carbenoid insertions using *spiro*-bisimine ligand **L25** at cryogenic temperatures (Scheme 39A). Ollevier, however, focused on making these reactions more general under ligand-free conditions at ambient temperature, and without the asymmetric component (Scheme 39B). The substrate scope was already broad, including both diazoesters and diazoketones as carbene precursors.

**Scheme 40:**
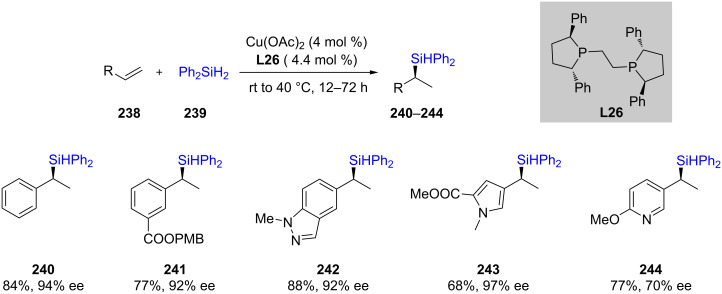
Enantioselective hydrosilylation of alkenes.

After the impressive report from Dow Corning on the hydrosilylation of alkenes in 2013 [78], recently, the Buchwald group extended this chemistry to the asymmetric hydrosilylation of alkenes [79]. They used nonracemic ligand (*S,S*)-Ph-BPE (**L26**) in combination with catalytic amounts of Cu(OAc)_2_ and stoichiometric Ph_2_SiH_2_ at ambient temperature to convert various alkenes to the desired enantiomerically pure silylated products (Scheme 40).

**Scheme 41:**
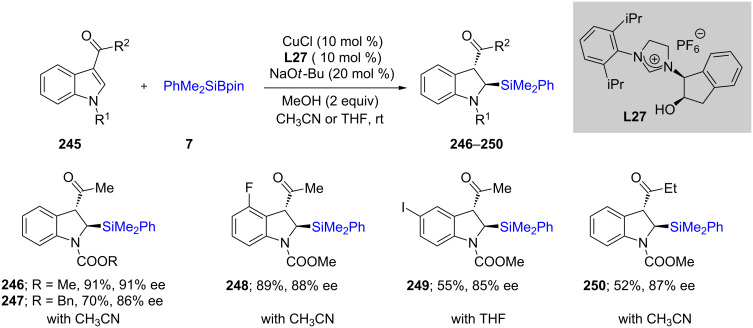
Conversion of 3-acylindoles to indolino-silanes.

**Scheme 42:**
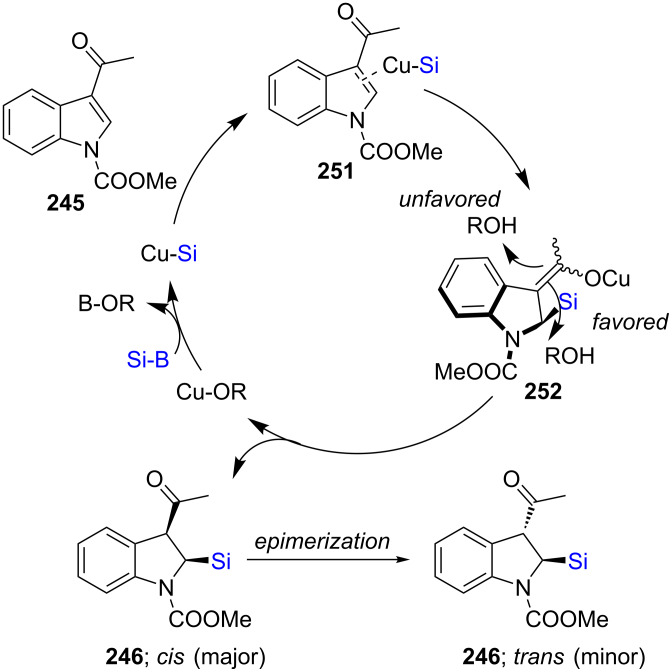
Proposed mechanism for the silylation of 3-acylindoles.

A highly regio- and enantioselective dearomative silyation of indoles using NHC **L27**–ligated CuCl has been disclosed recently [80]. A variety of 3-acylindoles **245** were converted to the desired indolino-silane products **246**–**250** in mostly moderate to good yields with very good enantioselectivities (Scheme 41). A mechanistic investigation revealed the importance of the acyl group at the 3-position. Systematic kinetic studies using NMR experiments suggested that protonation of the intermediate **252** occurs from the sterically favored side, leading to the kinetically stable *cis* product (Scheme 42). Nonetheless, some epimerization under the reaction conditions took place leading to the thermodynamically more stable *trans* product, **246**.

**Scheme 43:**
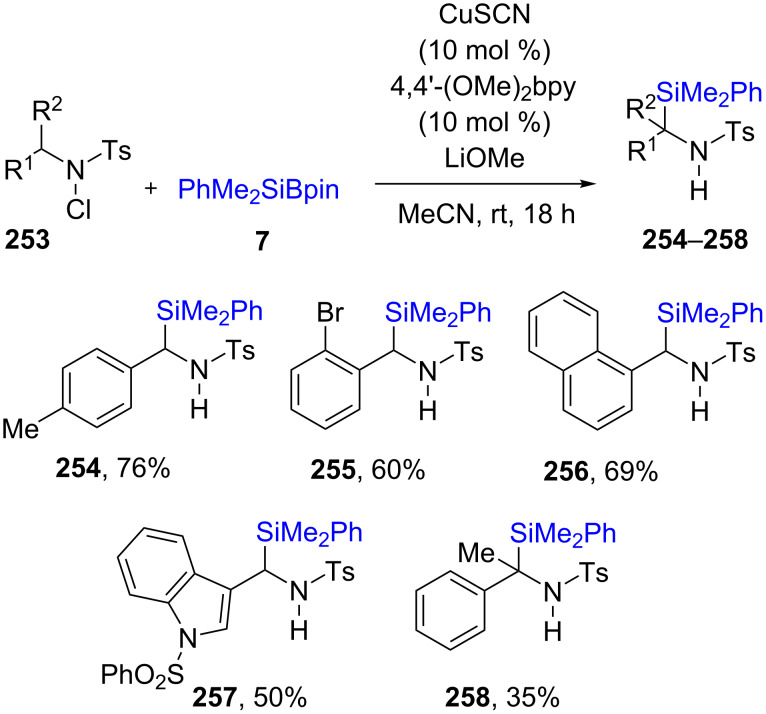
Silyation of *N*-chlorosulfonamides.

The direct activation of C(sp^3^)–H bonds attached to *N*-Cl tosylamines **253** was achieved via a radical pathway affording the products of silylation **254**–**258** in good chemical yields (Scheme 43) [81]. Most benzylic or benzylic-like positions are sufficiently activated to give tertiary carbon centers-bearing silicon products, although educts with initial tri-substitution led to lower isolated yields (e.g., **258**).

**Scheme 44:**
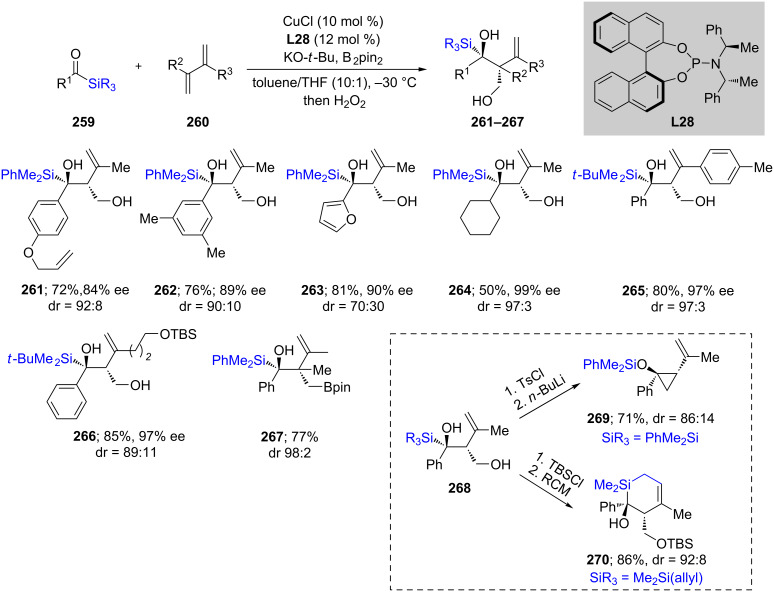
Conversion of acyl silanes to α-silyl alcohols.

An unusual type of reaction has been described in which an acyl silane reacts with 1,3-dienes, under Cu catalysis, leading to an interesting class of α-silyl tertiary alcohols (Scheme 44) [82]. In most cases, high chemical yields along with high ees were obtained when phosphoramidite ligand **L28** was used. A wide variety of compounds was prepared, including one with a bisquarternary center (**267**), although the Bpin residue within it could not be oxidized to the desired alcohol due to decomposition. Nonetheless, several products could be transformed into molecules of greater complexity. For example, cyclopropanation could be achieved to give **269**. Additionally, TBS protection of **268** followed by ring closing metathesis (RCM) led to the interesting 6-membered silacycle **270**.

**Scheme 45:**
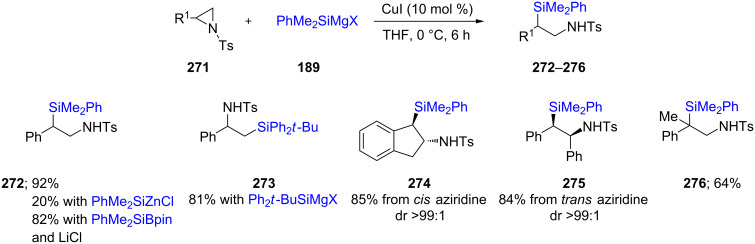
Conversion of *N*-tosylaziridines to β-silylated *N*-tosylamines.

This same research group recently reported on the addition of silyl Grignard reagents to aziridines under copper catalysis [83]. While the use of RMgX led to high chemical yields of the desired products, the corresponding catalytic Cu/zinc reagents gave poor yields (ca. 20%; Scheme 45). A library of aminosilane derivatives was prepared using this strategy to result in branched silyl compounds **272**–**276**. The addition of Grignard-derived copper reagents was stereospecific, where *cis-*aziridines gave *trans* products (e.g., **274**), and vice versa (e.g., **275**). The important aspect shown here is the utility of Suginome’s reagent along with LiCl, which completely overrode the need for a Grignard reagent and led to good chemical yields of the desired product (e.g., **272**). In one case examined, a bulky silyl Grignard reagent gave the linear silyl derivative selectively. In addition, a quaternary carbon bearing the PhMe_2_Si group could also be prepared in moderate yield (**276**).

**Scheme 46:**
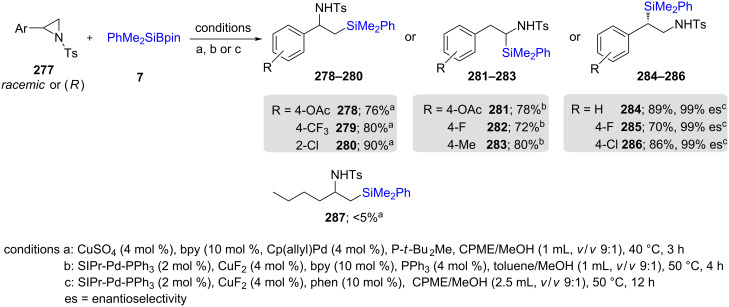
Conversion of *N*-tosylaziridines to silylated *N*-tosylamines.

The regiocontrolled ring opening reactions of the same aryl-substituted aziridines **277** have also been shown by Minakata and Takeda et al. to be susceptible to dual Pd/Cu catalysis. Depending upon the ligand on each metal, either the 2 or 3-position on the ring could be accessed. A dual catalytic cycle was proposed, where the Cu–Si species formed in situ undergoes transmetallation to the Pd(II) species resulting from the attack of Pd(0) on the aziridine ring, ultimately affording the silylated product with silicon at the benzylic site (Scheme 46). The product featuring the PhMe_2_Si residue at the β-location **287**, however, arises by way of a 1,2-addition to an imine, formed from the same Pd(II) intermediate via elimination [84].

**Scheme 47:**
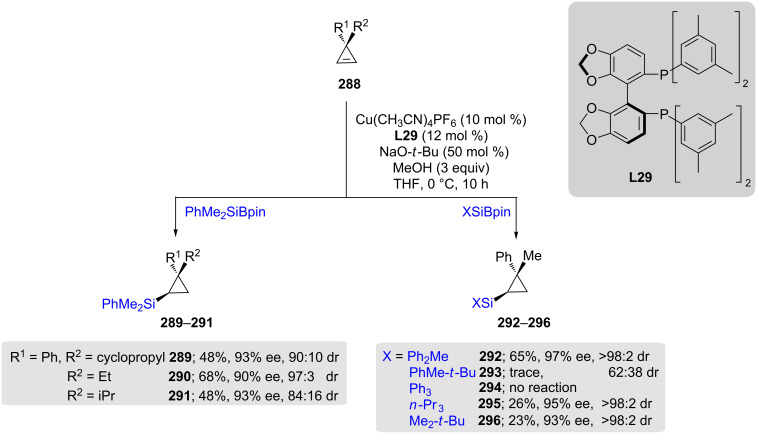
Conversion of 3,3-disubstituted cyclopropenes to silylated cyclopropanes.

Oestrich and co-workers have recently demonstrated non-directed, asymmetric *syn*-addition-silylations of 3,3-disubstituted cyclopropenes **288** using a Cu(I) pre-catalyst and (*R*)-DM-Segphos (**L29**) that take place with high enantio- and diastereoselectivities. They also studied the effect of geminal carbon substituents and found that as the bulk increases, both yields and diastereoselectivities decrease, while enantioselectivities remain unaffected. In addition to the impact of steric effects, variations in the alkyl-substituted silicon reagents also negatively impacted the chemical yields. However, again, there was no effect on enantioselectivity. Interestingly, upon replacement of the alkyl groups on the silicon by three phenyl rings **294** there was no conversion, highlighting the influence of the alkyl groups on silicon (Scheme 47) [85].

**Scheme 48:**
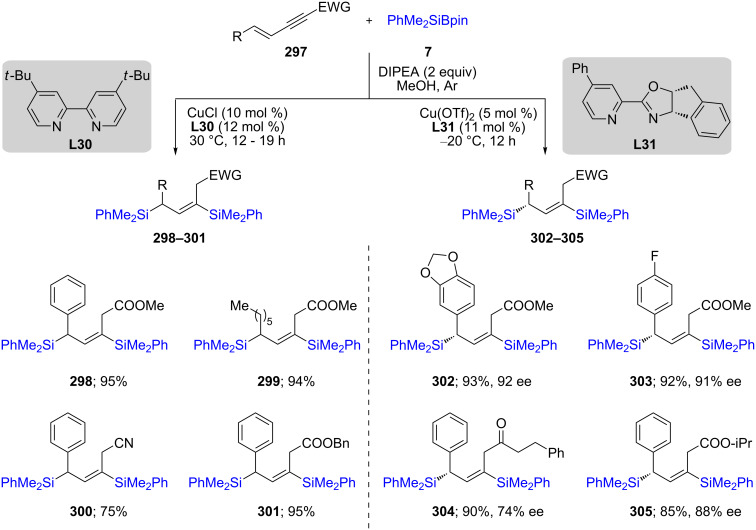
Conversion of conjugated enynes to 1,3-bis(silyl)propenes.

Xu and co-workers have described a simultaneous double silylation on conjugated enynes **297**, where either racemic or enantiomerically enriched 1,3-bis(silyl)propenes are formed in good yields (Scheme 48). They proposed a mechanism in which LCu(I)–Si coordinates first with the triple bond, which eventually forms a monosilylated diene. The resulting organocopper species then participates in a second catalytic cycle to furnish the disilylated products [86].

### 2 Cu-catalyzed carbon–boron bond formation

Organoboron compounds are widely used in C–C and C–X (X = N, O) bond constructions. Straightforward methods for their synthesis involve the copper-catalyzed addition of organoboron compounds to alkynes, alkenes, and unsaturated carbonyl compounds, as well as the nucleophilic borylation of alkyl or aryl halides. While there are reports on the formation of C–B bonds in the presence of NHC complexes [87] with Au [88], Pd [89], Pt [90], and Ir [91] catalysts, the focus here is on Cu-catalyzed reactions. Enantioselective processes have also been studied due to applications of medicinal interest involving optically active organoboron derivatives and their intermediacy as precursors to other functional groups, such as nonracemic alcohols. Achiral substrates in the presence of well-defined chiral copper complexes deliver chiral products, while nonracemic substrates can also react with achiral copper for similar purposes.

**Scheme 49:**
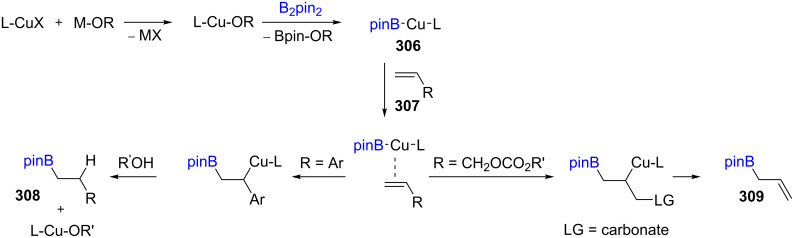
Proposed sequence for the Cu-catalyzed borylation of substituted alkenes.

In general, the pathway for introducing boron into unsaturated compounds (C–B coupling) mediated by a copper catalyst relies on the reaction of a Cu(I) salt with an alkoxide (M–OR) which then undergoes transmetallation with an organoborane to form, e.g., L-Cu-Bpin (**306**). This species serves as an active catalyst, nucleophilic at boron, via coordination with an alkene (**307**) which undergoes insertion to deliver the B–C bonded species via an intermediate that either undergoes elimination or reacts with an electrophile to form the product (e.g., **308**, **309**, Scheme 49) [92].

#### 2.1 Formation and reactions of allylic C–B bonds

**Scheme 50:**
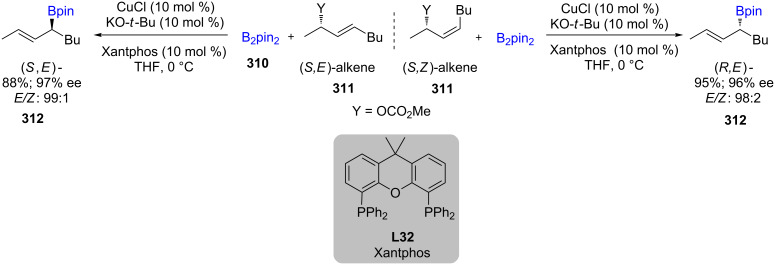
Cu-catalyzed synthesis of nonracemic allylic boronates.

The synthesis of α-stereogenic allylboronates was reported by Ito in 2005 using CuO-*t*-Bu/Xantphos (CuCl and KO-*t*-Bu form CuO-*t*-Bu in situ as the active catalyst precursor), an enantioenriched allyl carbonate, and B_2_pin_2_ (**310**; Scheme 50) [93]. Both (*Z*)- and (*E*)-alkenes **311** afforded the (*E*)-alkene **312** as the major product. The targeted γ-borylated compounds (relative to the leaving group) were formed, each with high enantioselectivity, which can be used for further stereoselective C–C and C–X (X = heteroatom) bond formation.

**Scheme 51:**
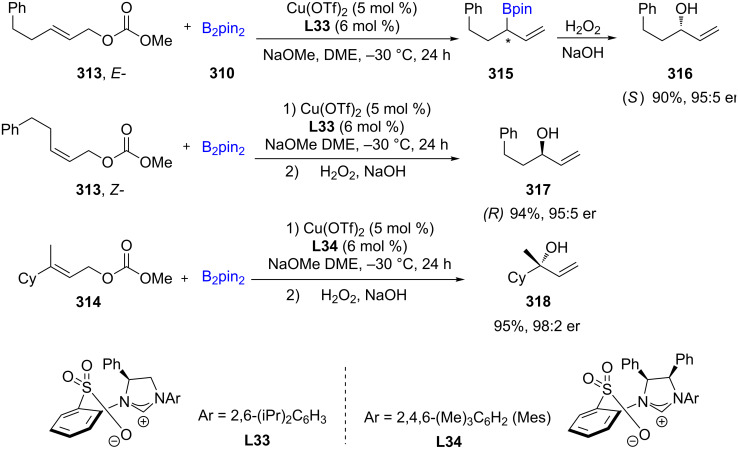
Cu–NHC catalyzed synthesis of α-substituted allylboronates.

Catalytic Cu(NHC)-mediated formations of enantioenriched α-substituted allylic boronates take place in high yields and site-selectivity (>98% S_N_2′) starting with either *trans*- or *cis*-disubstituted alkenes **313**, as well as linear or branched alkyl and aryl trisubstituted allylic carbonates **314**. The further oxidation of the boronated products (e.g., **316**) yielded nonracemic secondary (e.g., **317**) and tertiary alcohols (e.g., **319**). The presence of Cu(OTf)_2_, an imidazolium salt, and NaOMe leads to a chiral NHC–Cu complex, which, in the presence of B_2_pin_2_, generates the corresponding B–Cu species followed by its addition to allylic carbonates to deliver the targeted products (Scheme 51) [94].

**Scheme 52:**
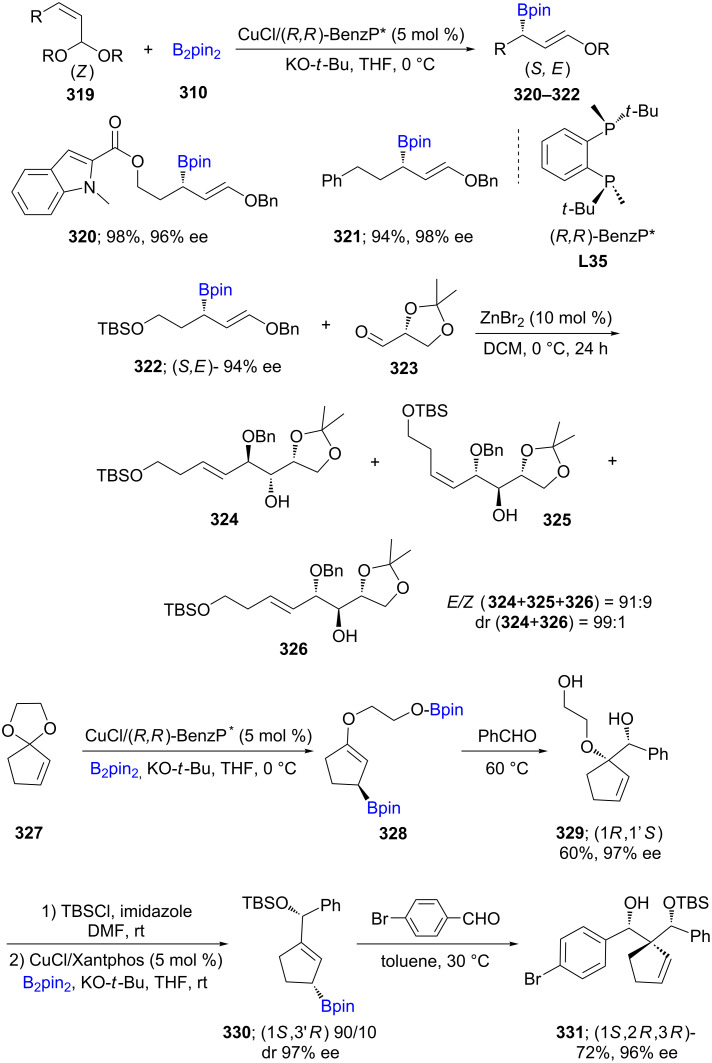
Synthesis of α-chiral (γ-alkoxyallyl)boronates.

In addition to allylic carbonates, allylic acetals (**319**) were also used for C–B bond formation that, in the presence of CuCl/(*R,R*)-BenzP* and stoichiometric amounts of KO-*t*-Bu, provide access to α-chiral linear or carbocyclic (γ-alkoxyallylic)boronates **320**–**322**. High functional group tolerance and enantioselectivities are characteristics of this reaction. The stereoselective formation of a 3,3-disubstituted cyclopentene scaﬀold (e.g., **331**), containing three contiguous asymmetric centers, was also developed starting from an achiral cyclic acetal **327**. In addition, the formation of an *anti*-1,2-diol with high enantioselectivity is also another outcome resulting from this protocol (Scheme 52) [95].

**Scheme 53:**
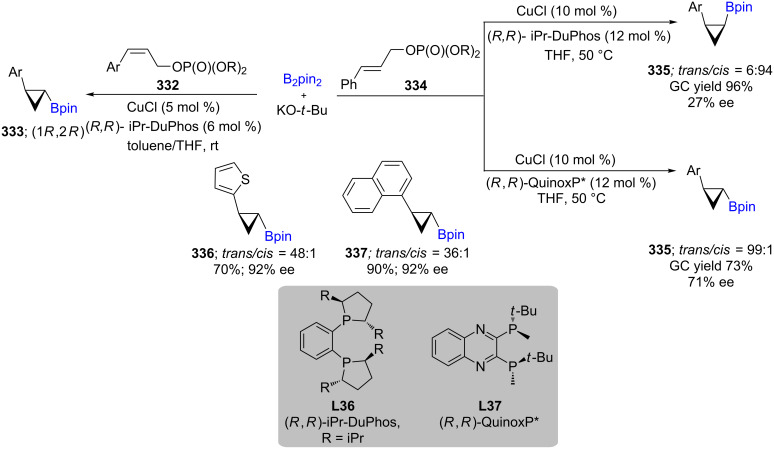
Cu-mediated formation of nonracemic *cis*- or *trans*- 2-substituted cyclopropylboronates.

Catalytic Cu-mediated conversions of (*Z*)-3-arylallylic phosphates **332** to nonracemic *trans*-2-aryl and -heteroaryl-substituted cyclopropylboronates **333** have been reported to take place in high yields, along with high diastereo- and enantioselectivities. Applications of optimized ligands, such as (*R,R*)-QuinoxP* and (*R,R*)-iPr-DuPhos, for borylation of (*E*)-allylic phosphates **334** delivers either the *trans* or *cis*-configuration of these cyclopropyl moieties (**335**; Scheme 53) [96].

**Scheme 54:**
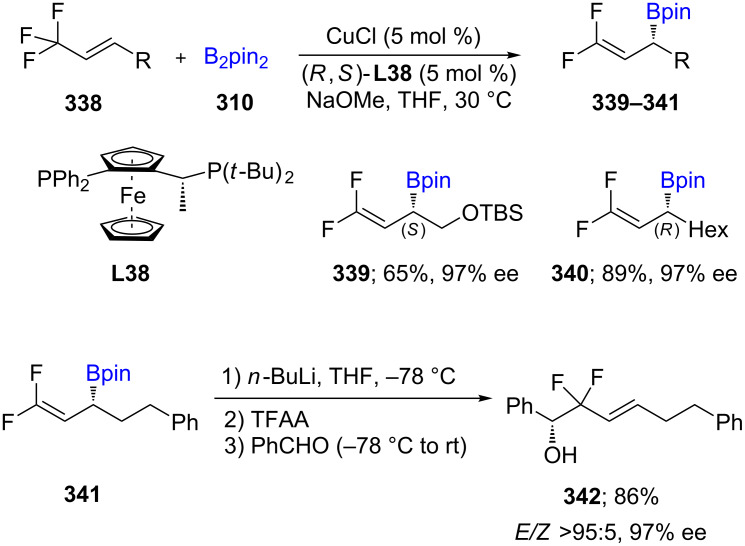
Cu-catalyzed synthesis of γ,γ-*gem*-difluoroallylboronates.

The first Cu(I)-catalyzed allylic displacement on alkenes containing a CF_3_ group was reported in 2018. Enantioenriched γ,γ-*gem*-difluoroallylic boronates **339**–**341** can be obtained via this process. Once the obtained difluoromethylene scaffolds are formed, they are readily converted to optically active secondary alcohols (e.g., **342**, Scheme 54) [97].

#### 2.2 Regioselective borylation of alkynes, alkenes, and allenes

Cu-catalyzed borylation of C–C multiple bonds involves the formation of nucleophilic Cu–B species that coordinate with a π-system to initially transfer the boryl group. This step is then followed by treatment of the reaction intermediate with an electrophile to deliver the desired borylated compound [98].

**Scheme 55:**
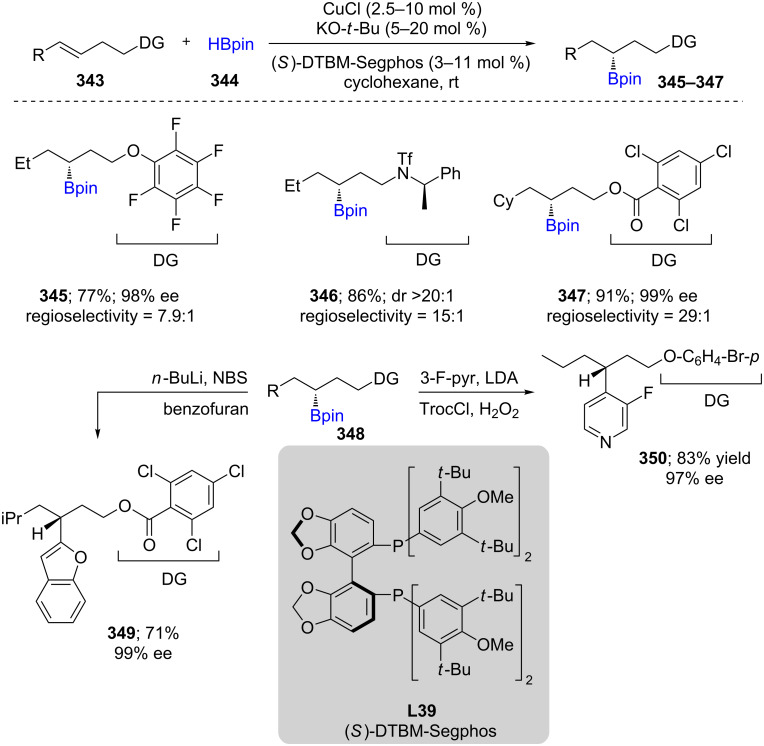
Cu-catalyzed hydrofunctionalization of internal alkenes and vinylarenes.

An efficient Cu-catalyzed (via in situ formed [(*R*)-DTBM-Segphos]CuH) protocol for an asymmetric net hydroboration of internal alkenes **343** with high regio- and enantioselectivity was reported by Hartwig et al. in 2016 [99]. The newly formed C–B bond reacted with a range of electrophiles to deliver products containing C–C, C–N, and C–X (X = Br, Cl) bonds (e.g., **349**, **350**). Efforts to explore the mechanism revealed a decrease in regioselectivity when the C=C bond is further from the directing group. This was attributed to the importance of both the negative charge at the carbon and the forming C–Cu bond being developed in the transition state, which is stabilized by the positive charge on the carbon bearing the directing group. Later, in 2017, the same group investigated the details of the mechanism for the hydrofunctionalization of internal alkenes and vinyl arenes (Scheme 55) [100]. Unlike acyclic alkenes, Tortosa et al. utilized cyclobutenes as well as bicyclic cyclobutenes as educts using (*R*)-DM-Segphos for Cu-catalyzed enantioselective borylation, affording cyclobutyl boronates in high yields and with excellent stereocontrol [101]. This work followed on the heels of earlier studies on the borylation of 2,6-disubstituted *p*-quinone methides en route to enantioenriched mono- and dibenzylic boronates [102].

**Scheme 56:**
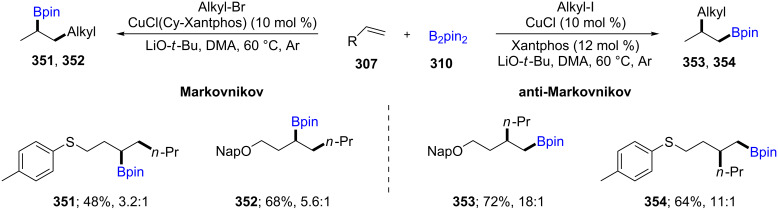
Cu-catalyzed Markovnikov and *anti*-Markovnikov borylation of alkenes.

An efficient regio-divergent method starting from a single alkene **307** (e.g., protected allylic alcohols) was explored by varying the ligand on copper. Using catalytic CuCl/Xantphos, direct access to *anti*-Markovnikov alkylborated products (e.g., **353**, **354**) was noted. Alternatively, simply switching from Xantphos to Cy-Xantphos afforded the products of Markovnikov addition (e.g., **351**, **352**). It was shown that the presence of a heteroatom plays a crucial role due to the otherwise non-selective, facile addition of Cu-Bpin to alkenes (Scheme 56) [103]. Based on previous studies on asymmetric 3-component carboboration of styrene derivatives [104] and 1,3-dienes [105] with C(sp^2^)-containing electrophiles such as *tert-*butyl allyl carbonate or aldimines using B_2_pin_2_, Liao et al. reported the same approach, albeit using more challenging C(sp^3^) electrophiles (e.g., CH_3_I) for the enantioselective methylboration. Both aliphatic alkenes and styrenes could be used with either CuCl/quinoxP* or their in-house-developed chiral sulfoxide phosphine ligand (SOP). Excellent diastereo- and enantioselectivities were obtained. A gram scale synthesis of (*S*)-naproxen was also described as a “real world” application [106].

**Scheme 57:**
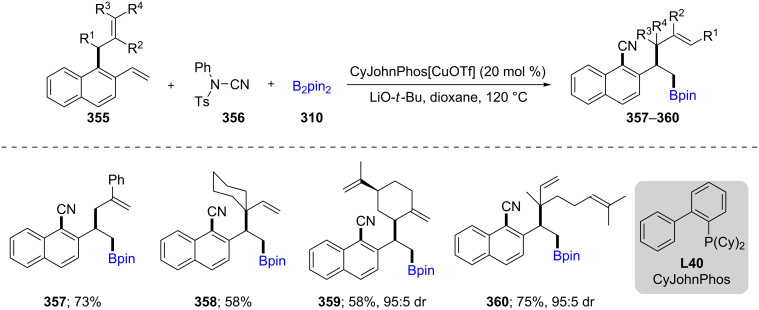
Cu-catalyzed borylation/ortho*-*cyanation/Cope rearrangement.

From previous findings involving trapping of a vinylarene-derived benzylic copper species with an electrophilic source of a cyano residue, Yang and co-workers reported on the Cu-catalyzed borylation of styrenes bearing an allylic group at the 1-position. After the initial addition, a cascade of reactions occurred, including cyanation generating a dearomatized intermediate. This species then undergoes [3,3]-sigmatropic rearrangement, positioning the allylic unit in a regio- and stereospecific manner, along with rearomatization to afford the products **357**–**360** (Scheme 57) [107].

**Scheme 58:**
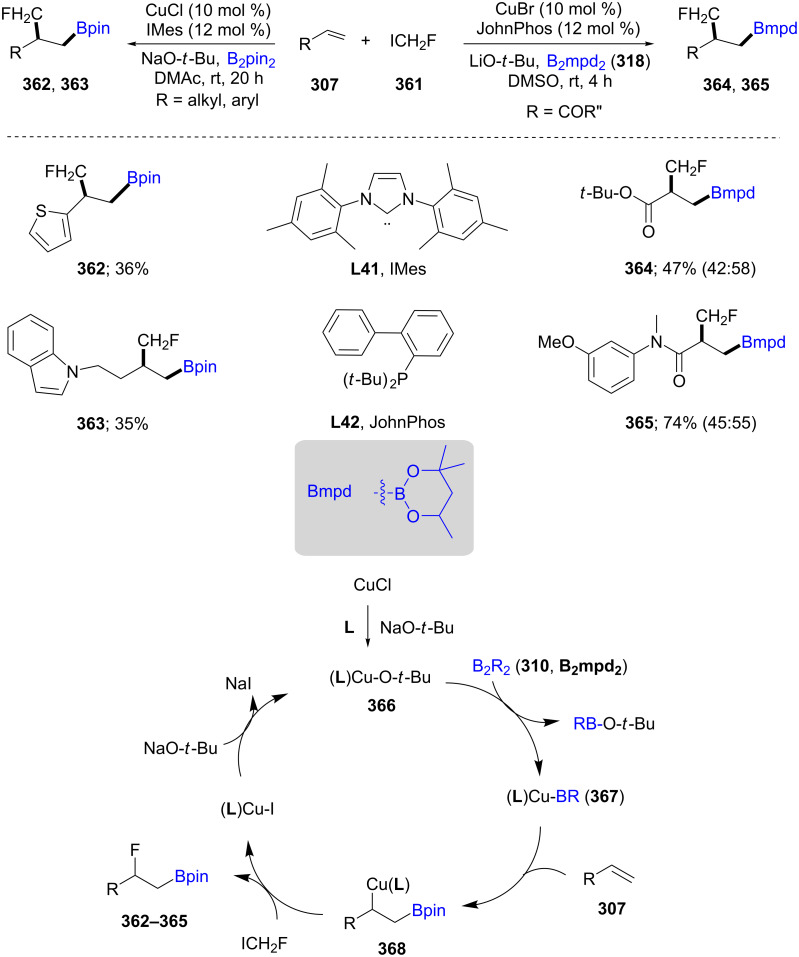
Borylﬂuoromethylation of alkenes.

The borylﬂuoromethylation of acrylamides, acrylates, and heteroaromatic-substituted alkenes (**307**) was established using CuBr/JohnPhos/LiO-*t*-Bu and CuCl/IMes/NaO-*t*-Bu, respectively, in the presence of I-CH_2_F (**361**) as the methylfluorinating agent, affording products reflecting excellent control of regioselectivity. This transformation is useful for the synthesis, e.g., of monoﬂuorinated ibuprofen, through sequential oxidation of the product with NaBO_3_·4H_2_O and Jones reagent. The catalytic cycle includes formation of (L)Cu-O-*t*-Bu (**366**) which then reacts with B_2_R_2_ (**310**, B_2_mpd_2_) to form (L)Cu–BR (**367**). The reaction of this species with the olefin **307** afforded a borylcuprated intermediate **368**, which upon reaction with I-CH_2_F leads to the borylﬂuoromethylated product (e.g., **362**–**365**, Scheme 58) [108].

**Scheme 59:**
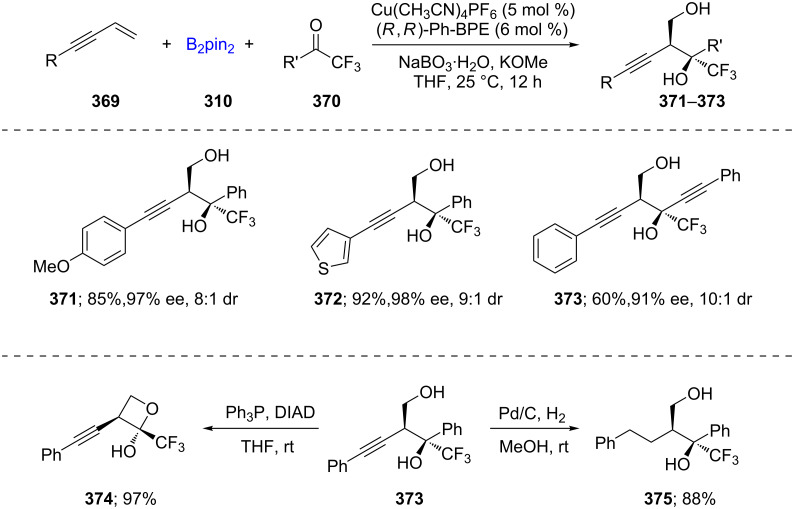
Cu-catalyzed synthesis of tertiary nonracemic alcohols.

Yin and co-workers described Cu(I)-catalyzed asymmetric additions to challenging trifluoromethyl and perfluoroalkyl ketones **370**, starting with 1,3-enynes **369** and a slight excess of B_2_pin_2_ in THF at room temperature. The initial homopropargylic borane adduct, upon oxidation with NaBO_3_·H_2_O, yields the desired nonracemic tertiary alcohols **371**–**373** in typically high chemical yields, good diastereoselectivities, and impressive enantioselectivities. The subsequent treatment of these newly formed adducts (e.g., **373** to form products, **374** and **375**; Scheme 59) highlights the utility of this methodology [109].

**Scheme 60:**
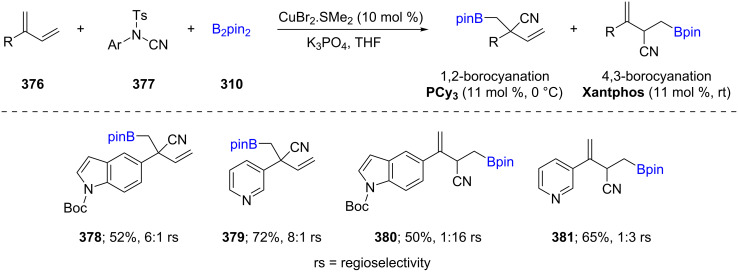
Synthesis of densely functionalized and synthetically versatile 1,2- or 4,3-borocyanated 1,3-butadienes.

Following Hoveyda’s report on enantioselective Cu-catalyzed conjugate additions of borylated butadiene, generated in situ, to enolates [110], Procter et al. described the regio-divergent conversion of 2-substituted 1,3-dienes **376** into borocyano products using commercially available phosphine ligands (Scheme 60). Simply switching from a mono to a bidentate ligand resulted in 1,4 to 4,1-borocupration. The formation of allyl–Cu intermediates in the catalytic cycle readily trap available electrophiles, such as *N*-cyanosulfonamides, leading to 1,2 or 4,3-borocyanation in a regiospecific fashion from borocuprative intermediates, respectively [111].

**Scheme 61:**
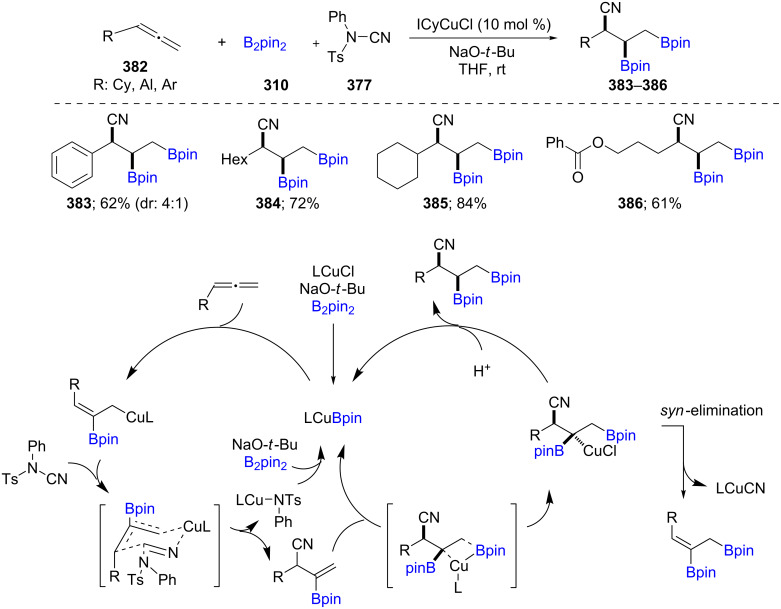
Cu-catalyzed trifunctionalization of allenes.

Extending earlier efforts from the Montgomery group for Cu-catalyzed cascade diborylation/ortho-cyanations of terminal allenes [112] and styrene derivatives [113], the cyanoborylation of terminal allenes **382** has also been studied. Using B_2_pin_2_ in the presence of *N*-cyano-*N*-phenyl-*p*-toluenesulfonamide (NCTs, **377**), under Cu-catalysis led to regio-, chemo-, and diastereoselective trifunctionalized products **383**–**386**. The reaction sequence involves three steps: first, borocupration followed by an electrophilic cyanation, and finally, a second borocupration. It was discovered that steric factors determine the site of the first borocupration, while electronic effects are dominant in the second addition of boron (Scheme 61) [114].

**Scheme 62:**
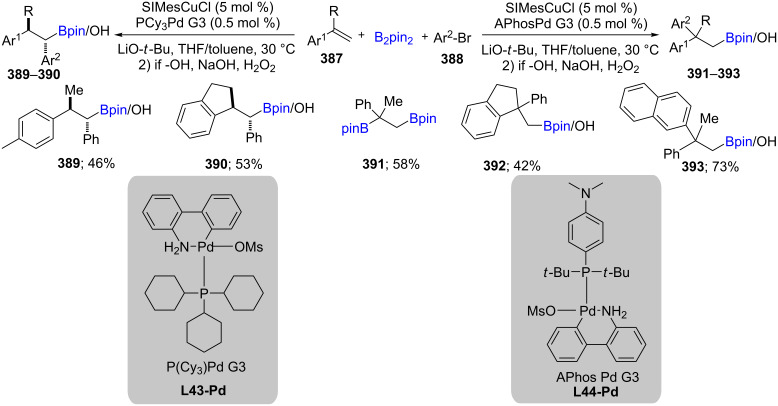
Cu-catalyzed selective arylborylation of arenes.

The selective arylborylation of α-alkylstyrenes **387** has been investigated, delivering 1,1-adducts **389**, **390** in the presence of palladacycle PCy_3_Pd G3, while 1,2-adducts **391**–**393** result using APhosPd G3. The former reaction proceeds through a second-stage β-hydride elimination/re-insertion pathway, although for the latter set of conditions, a rare cross-coupling takes place (Scheme 62) [115].

The cooperative effect of a catalytic system consisting of Cu/Ni (e.g., Ni(acac)_2_, CuCl, and PCy_3_) was also reported by Nakao et al. leading to regio- and stereoselective arylboration of 1-arylalkenes with aryl chlorides or tosylates. The reactions tolerate a variety of functional groups (including silyl ether, alkoxycarbonyl, and aminocarbonyl) [116]. Following the expected mechanistic sequence, Brown et al. generated a variety of Cu-catalyzed carboborylated products from *trans-*β-substituted styrenes [117] and *cis-*β-substituted styrenes [118] coupled with aryl/heteroaryl halides using SIMesCuCl/Pd-RuPhos G3 to give the *syn*-isomers, while SIMesCuCl/Pd-P-iBu_3_ gave the *anti*-isomers. Styrenes could also be coupled with acyl chlorides [119] as electrophiles in the presence of B_2_(pin)_2_. Likewise, these same authors coupled 1,2-disubstituted 1,3-dienes with 3-bromopyridine derivatives and B_2_(pin)_2_ under both racemic and non-racemic conditions and short reaction times [120].

**Scheme 63:**
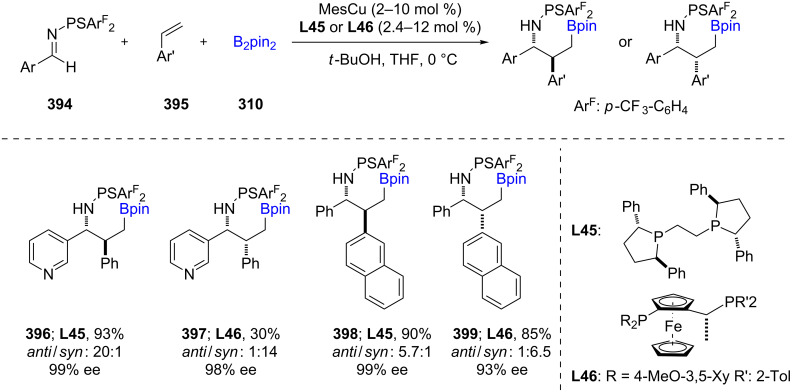
Asymmetric borylative coupling between styrenes and imines.

Following various reports [121–122] of nonracemic borylative coupling of alkenes with imines, Kanai and coworkers [123] demonstrated a related enantio- and diastereo-divergent borylative coupling of styrenes **395** with *N*-phosphinoyl and *N*-thiophosphinoyl imines **394** to afford α,β-disubstituted γ-borylated nonracemic amines **396**–**399**. A combination of CuMes with either (*R*,*R*)-Ph-BPE or (*R*)-(*S*_p_)-Josiphos afforded the targeted products in high ees (Scheme 63). The asymmetric addition of benzylic–Cu intermediates to imines proceeds through a flexible linear transition state which is sensitive to the steric environment surrounding the copper catalyst. This observation provided the opportunity to achieve either diastereomer depending on the choice of the ligand **L45** or **L46**.

**Scheme 64:**
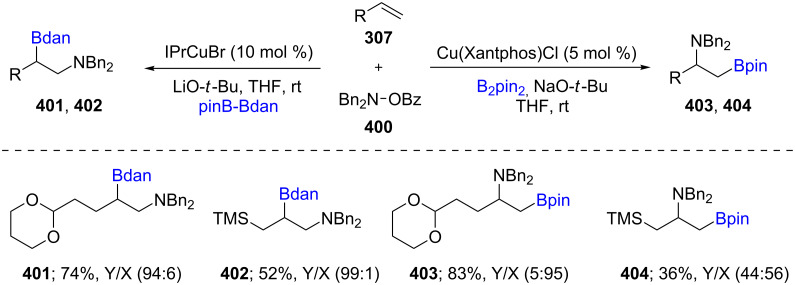
Regio-divergent aminoboration of unactivated terminal alkenes.

Based on the interest in Cu-catalyzed aminoboration reactions of a variety of alkenes, such as bicyclic alkenes [124], Miura et al. subsequently described the preparation of β-borylalkylamines **401**–**404** from unactivated terminal alkenes **307** (Scheme 64). Using a mixed diborane reagent, such as pinB–Bdan developed by Suginome [125], or B_2_pin_2_, along with various *N*-hydroxylamine derivatives (**400**) under ligand-influenced Cu catalysis, high regioselectivities were typically obtained [126]. A year later, Popp et al*.* reported a Cu-catalyzed regiospecific boracarboxylation of vinyl arenes using 1 atm CO_2_ and B_2_pin_2_ in moderate to excellent yields [127].

#### 2.3 β-Borylation of α,β-unsaturated compounds

**Scheme 65:**
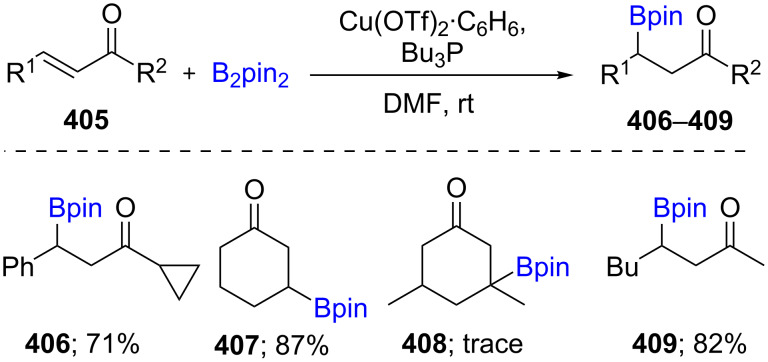
Cu-catalyzed 1,4-borylation of α,β-unsaturated ketones.

The use of an inexpensive transition metal like copper as catalyst is attractive for β-borylations of α,β-unsaturated carbonyl compounds, that can then be further functionalized. In 2000, an initial report featuring a 1,4-borylation of α,β-unsaturated compounds **405** was reported by Hosomi in the presence of Cu(OTf)_2_·C_6_H_6_/(*t*-Bu)_3_P in DMF. No reaction occurred using (CuOTf)_2_·C_6_H_6_ in the absence of (*t*-Bu)_3_P. Mechanistic studies showed that the coordination of phosphorus(III) to Cu(I) accelerated the reaction, as was confirmed by ^31^P NMR. Using equimolar diboron (e.g., B_2_pin_2_) slightly changed the chemical shift of (*t*-Bu)_3_P from −30.7 ppm to −31.4 ppm. In addition, a significant downfield shift was observed in the presence of CuCl/(*t*-Bu)_3_P (−14.2 ppm). Therefore, it can be concluded that the extent of coordination of (*t*-Bu)_3_P to CuCl is far greater than that with the diboron species, which results in an enhancement of the catalytic activity (Scheme 65) [128].

**Scheme 66:**
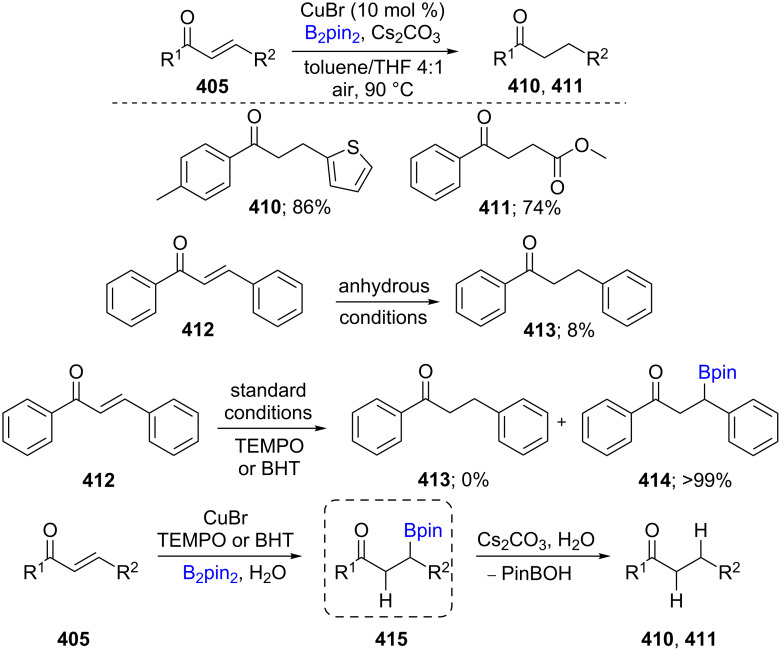
Cu-catalyzed protodeboronation of α,β-unsaturated ketones.

The one-pot borylation/protodeboronation of α,β-unsaturated ketones **405** in the presence of CuBr, B_2_pin_2_ and H_2_O as the hydrogen source was developed under mild reaction conditions to yield saturated ketones **410** and **411** in good to excellent yields. Hydrogen isotope labelling showed that water was likely the source of hydrogen since no reaction was observed under anhydrous conditions. By contrast, the addition of either TEMPO and BHT to the reaction resulted in 99% of the borylated product **414**, which may indicate the presence of a radical intermediate in the second, protodeboronation step. Therefore, the key intermediate is the 1,4-adduct **415**, which in the presence of base and water affords the saturated product (e.g., **410**, **411**; Scheme 66) [129].

**Scheme 67:**
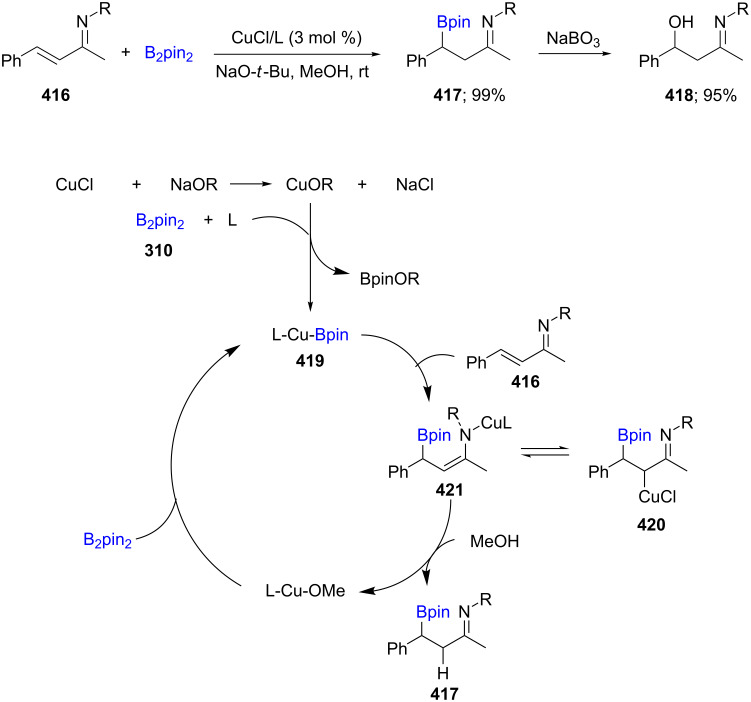
Cu-catalyzed β-borylation of α,β-unsaturated imines.

The β-borylation of α,β-unsaturated imines **416** was reported using CuCl/L/NaO-*t*-Bu (L = PCy_3_, Ph_3_P, JosiPhos) which, upon oxidation with NaBO_3_, delivered the desired β-imino alcohol **418** (Scheme 67). It is postulated that the base is responsible for the displacement of chloride from the catalyst and also cleavage of the B–B bond in B_2_pin_2_ to form the borylcopper intermediate. Following this, the metallo-enamine is formed before reacting with an electrophile [130].

**Scheme 68:**
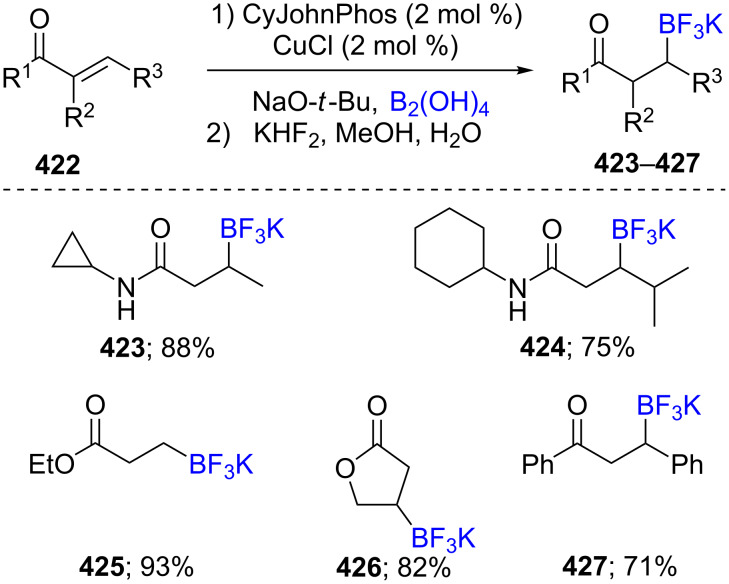
Cu-catalyzed synthesis of β-trifluoroborato carbonyl compounds.

A method to prepare β-trifluoroborate salts **423**–**427** each containing a carbonyl group was developed using a combination of CuCl/CyJohnPhos/NaO-*t*-Bu as the catalytic system. The conditions employed B_2_(OH)_4_ as an atom economical source of boron, starting from α,β-unsaturated amides **422**, ketones, and esters in MeOH as the solvent (Scheme 68) [131]. The β-amidotrifluoroborates synthesized through this protocol do not encounter the purification difficulties typically associated with conversions of pinacol esters to trifluoroborates.

**Scheme 69:**
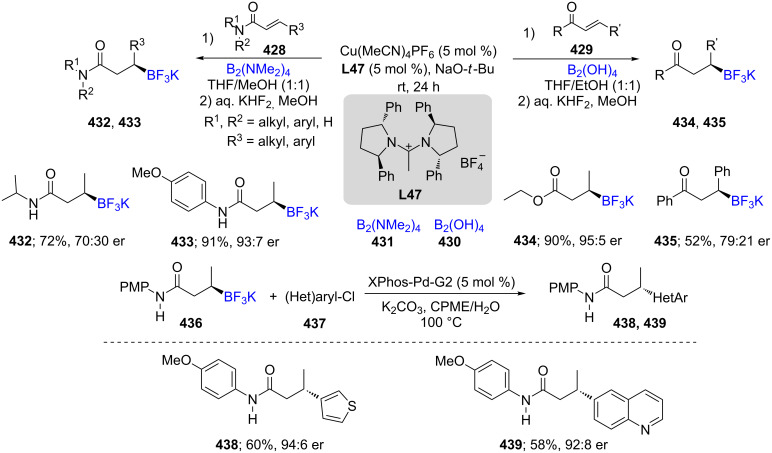
Asymmetric 1,4-borylation of α,β-unsaturated carbonyl compounds.

An asymmetric 1,4-borylation of α,β-unsaturated carbonyl derivatives, e.g., **428** and **429**, was reported using a combination of Cu(MeCN)_2_PF_6_/**L47** with either bisboronic acid (BBA, **430**) or tetrakis(dimethylamino)diboron (**431**) as atom-economical boron sources leading to the desired products **432**–**435** in moderate to good ees (Scheme 69) [132]. In addition, the reaction of potassium *N*-(4-methoxyphenyl)-3-(trifluoroborato)butanamide (**436**) as the nucleophilic coupling partner was investigated with heteroarylchlorides **437** leading to products reflecting a complete inversion of stereochemistry. The screening showed that XPhos-Pd-G2 is the best pre-catalyst for these reactions and it was proposed that the reaction takes place through an S_E_2 pathway due to coordination of the carbonyl group of the nucleophile to the boronate intermediate.

**Scheme 70:**
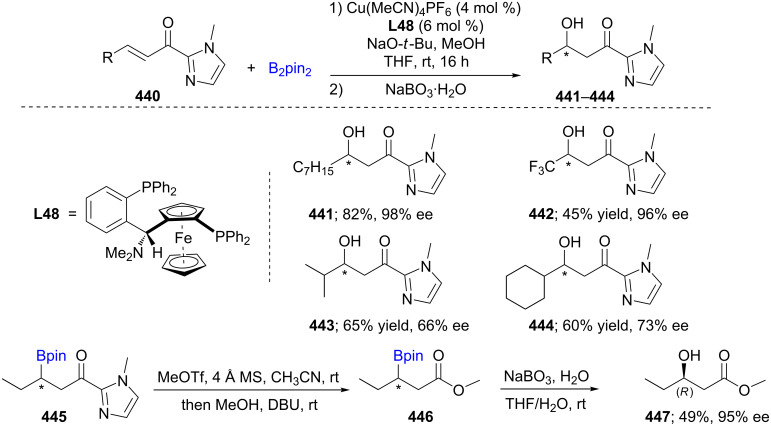
Cu-catalyzed ACB and ACA reactions of α,β-unsaturated 2-acyl-*N*-methylimidazoles.

Very recently the asymmetric Cu-catalyzed conjugate borylation (ACB) and addition (ACA) reactions onto α,β-unsaturated 2-acyl-*N*-methylimidazoles **440** have been developed using nonracemic Taniaphos (**L48**) as ligand, followed by an oxidation to the secondary alcohols resulting in high enantioselectivities. Aliphatic chains gave a high degree of enantioselectivity (up to 98%), while more moderate ees were noted when the substrates were branched at the γ-site (Scheme 70) [133]. The absolute configuration at each stereogenic center was determined by subsequent conversion of the borylated acylimidazole **445** to the corresponding ester **446**, which then was reacted with NaBO_3_ to deliver the nonracemic hydroxy derivative **447**.

#### 2.4 Nucleophilic borylation of C=X bonds

**Scheme 71:**
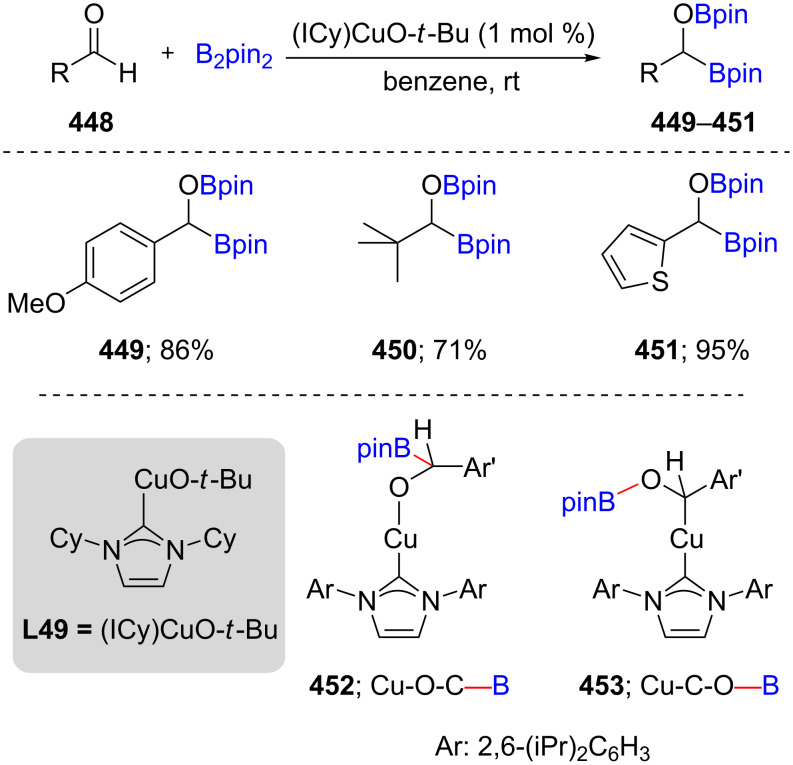
Cu-catalyzed diborylation of aldehydes.

The first studies on the borylation of both aliphatic and aromatic aldehydes **448** were carried out using Cu/NHC-ligand complexes generating diborylated products **449**–**451**; Scheme 71, top) [134]. The insertion of an in situ-formed Cu–B species into the aldehyde carbonyl was proposed, leading to the formation of a C–B bond. ^1^H NMR studies, however, did not confirm the formation of this Cu–O–C–B linkage **452**. Instead, solely the Cu–C–O–B species **453** was observed. Due to the possibility of facile rearrangement of the former to the latter, it could not be concluded that a direct insertion of the aldehyde into the Cu–B bond is taking place.

**Scheme 72:**
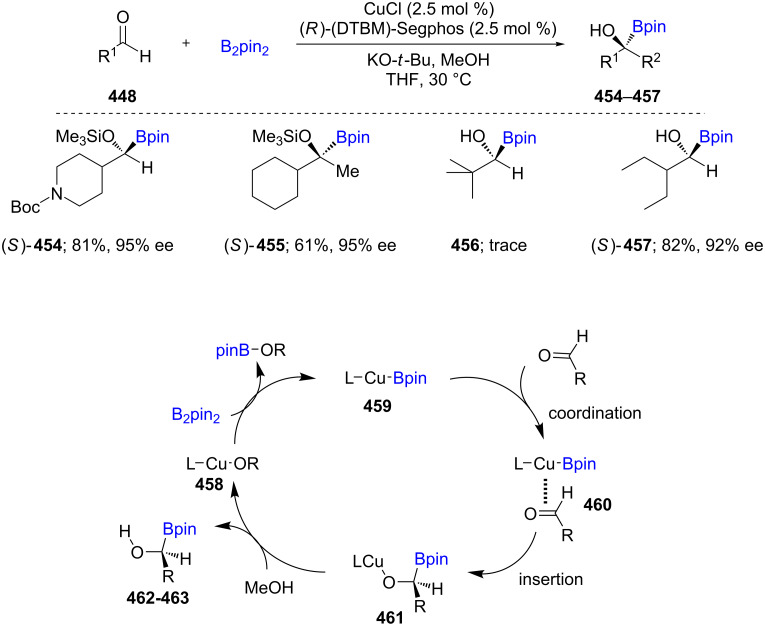
Umpolung pathway for chiral, nonracemic tertiary alcohol synthesis (top) and proposed mechanism for product formation of **456** and **457** (bottom).

The enantioselective borylation of aliphatic aldehydes **448** was first reported in 2015 using CuCl/KO-*t*-Bu/(*R*)-(DTBM)-Segphos as the active catalyst in THF for the formation of chiral, nonracemic α-alkoxyorganoboronate esters (up to 99% ee; **454**–**457**). MeOH was used as the proton source. Further functionalization can be considered as an umpolung pathway for the formation of enantioenriched tertiary alcohols through subsequent C–C bond formation (Scheme 72) [135]. The proposed mechanism involves the formation of L–Cu–O–*t*-Bu **458** which reacts with B_2_pin_2_ to generate L-Cu-Bpin **459**. The subsequent coordination to the aldehyde results in the intermediate **461**. Upon protonation of **461** the desired enantiomerically enriched borylated products **462** and **463** are obtained.

**Scheme 73:**
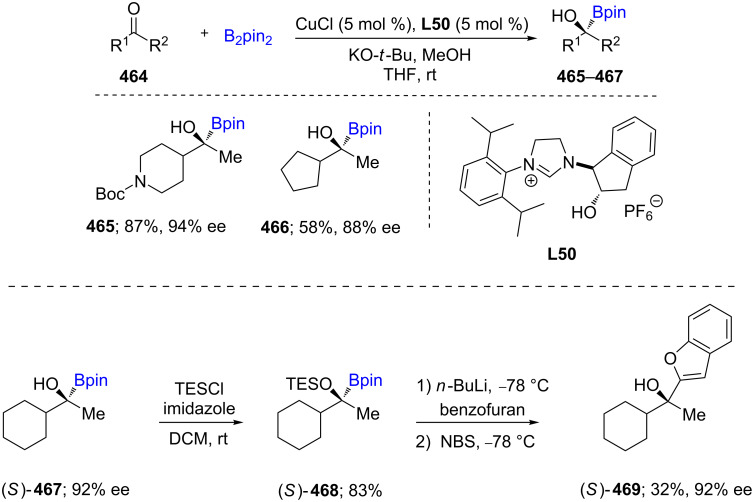
Cu-catalyzed synthesis of α-hydroxyboronates.

In 2017, a CuCl/nonracemic NHC combination of reagents was employed as catalyst leading to an efficient enantioselective formation of α-hydroxyboronates **465**–**467** in up to 94% ee from aliphatic ketones **464**. The subsequent treatment with *n*-BuLi followed by an appropriate electrophile led to C–C bond formation, ultimately delivering chiral tertiary alcohols. Mechanistic studies and DFT calculations showed that an in situ*-*formed borylcopper(I) species is responsible for the 1,2-addition (Scheme 73) [136].

**Scheme 74:**
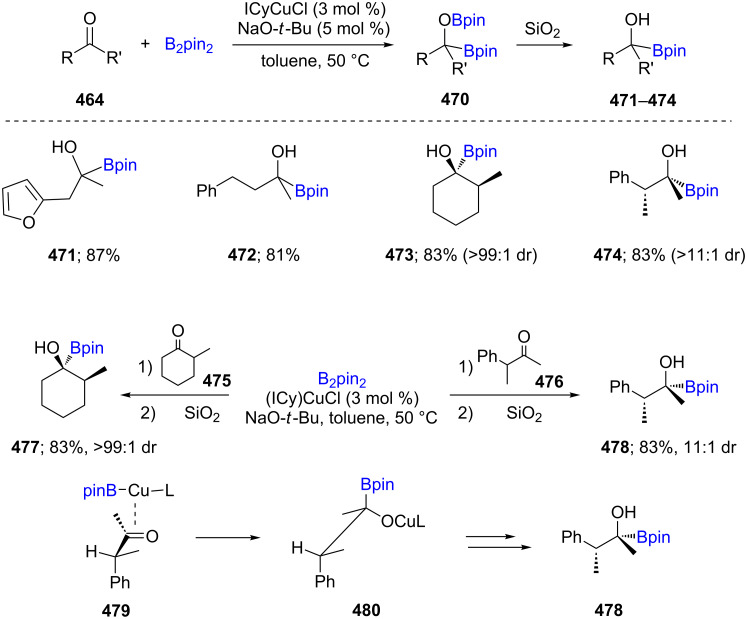
Cu-catalyzed borylation of ketones.

C,O-Diboration of ketones **464** was explored using a catalytic system consisting of (ICy)CuCl/NaO-*t*-Bu (ICy = *N,N*-dicyclohexylimidazolyl) in toluene which afforded the products **470** in moderate to high yields. Subsequently, tertiary α-hydroxyboronate esters **471**–**474** were isolated through O–B bond cleavage upon treatment with silica (Scheme 74) [137]. The diastereoselective formation of α-hydroxyboronate esters **477** and **478** starting from cyclic and acyclic ketones **475** and **476**) arises from an extensive steric crowding during the insertion reaction of the coordinated L-Cu-Bpin to the aldehyde **479**. The observed selectivity from Felkin-Anh-controlled addition to the carbonyl is increased here due to steric congestion associated with the Bpin substituent.

#### 2.5 Borylation of C–X bonds

**Scheme 75:**
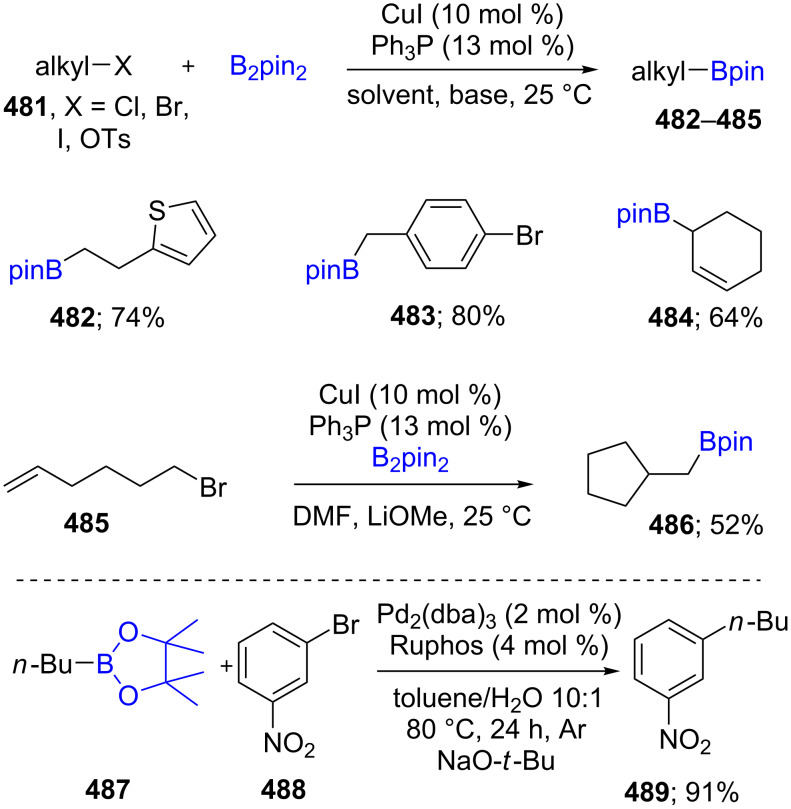
Cu-catalyzed borylation of unactivated alkyl halides.

Decades ago, both aryl or vinyl boronates were synthesized via Pd-catalyzed Miyaura borylations using aryl or vinyl halides as substrates, respectively [138]. Unactivated alkyl halides (X = Cl, Br, I, OTs; **481**), however, have been examined far more recently under mild conditions, using a combination of B_2_pin_2_ together with CuI/Ph_3_P and Li-OMe. Those alkyl halides bearing a double or triple bond in an appropriate position deliver cyclized borylated products **486**. Hence, both primary and secondary boronates could be obtained with high functional group tolerance, which is hard to access through previous protocols. Moreover, the desired alkylboronates **487** can be used further for Suzuki–Miyaura coupling reactions with aryl bromides **488** utilizing Ruphos as the ligand on palladium (Scheme 75) [139].

**Scheme 76:**
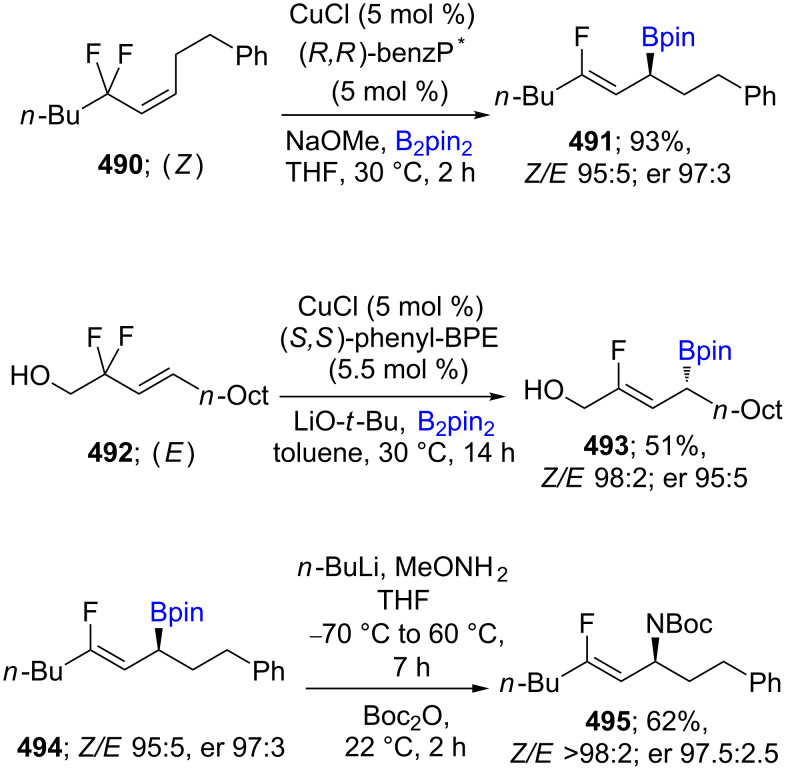
Cu-catalyzed borylation of allylic difluorides.

Early in 2019, a highly diastereo- and enantioselective conversion of trisubstituted alkenyl difluorides, where both *Z*- and *E*-olefinic isomers **490** and **492** afforded high levels of *Z*-olefinic boronates **491** and **493** was described. However, the *Z*-configured alkenes together with the catalyst system consisting of CuCl/BenzP* gave the *R*-configured alkylboronates, while a change in the ligand to the BPE ((+)-1,2-*bis*((2*S*,5*S*)-2,5-diphenylphospholano)ethane) series, led to the opposite isomer at the newly formed C(sp^3^) center. These fluorine-containing borylated products can be further converted to allylic amines (**495**; Scheme 76) [140].

**Scheme 77:**
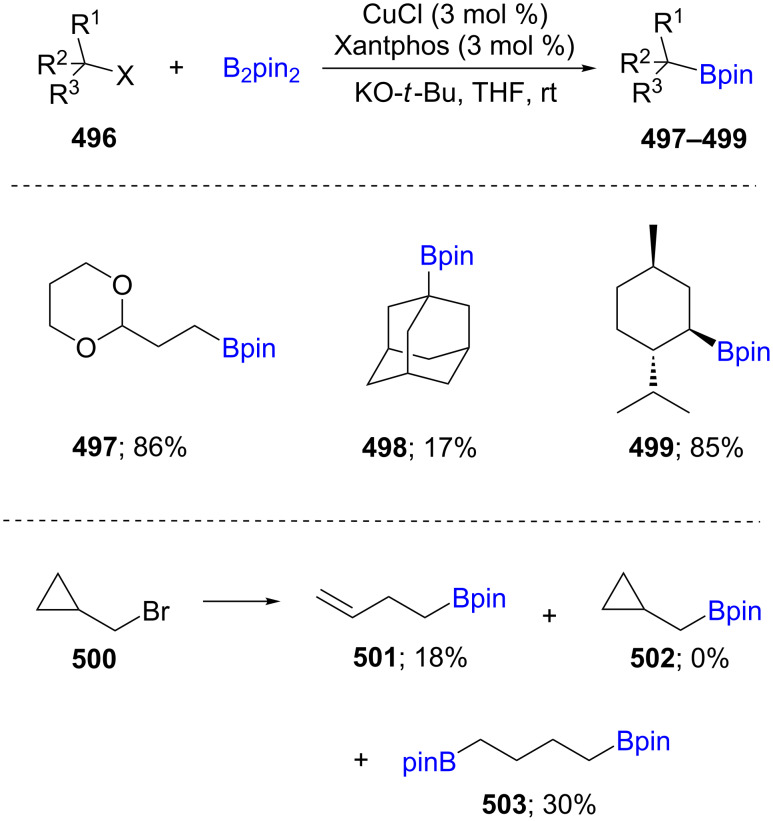
Cu-catalyzed borylation of cyclic and acyclic alkyl halides.

Borylation of cyclic and acyclic alkyl halides (X = Cl, Br, I; **496**) was developed using a catalyst derived from CuCl and the ligand Xantphos, with stoichiometric amounts of KO-*t*-Bu in one pot. No reaction was reported, however, using alkyl mesylates. Ring-opened products were observed using cyclopropylmethyl bromide (**500**) which suggests a radical pathway. A variety of functional groups was tolerated, and high diastereoselectivity of the newly formed products was maintained; e.g., in the case of menthyl halides (**499**; Scheme 77) [141].

**Scheme 78:**
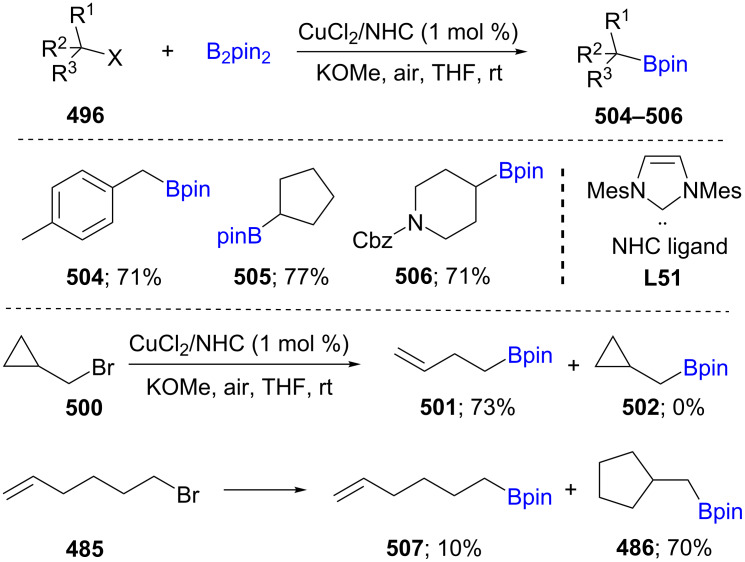
Cu-catalyzed borylation of unactivated alkyl chlorides and bromides.

A novel protocol was developed for the borylation of unactivated alkyl chlorides **496** and bromides using catalytic Cu(II) together with NHC **L51**. Similar to mechanistic studies for catalytic Cu(I) reactions, these Cu(II)–NHC complexes may activate B_2_pin_2_ forming a copper–boryl species, which further reacts with an alkyl halide as electrophile. The reactions, which could be performed under air, provide the desired borylated products in good yields while tolerating a wide range of functional groups. The reaction of cyclopropylmethyl bromide (**500**) leads to the ring-opened product, while in the case of 6-bromohex-1-ene (**485**), the cyclic alkylboronate (**486**) was formed as the major product. Both results suggest a radical pathway (Scheme 78) [142]. Enantioenriched benzylboronates were also synthesized from racemic benzyl chlorides using Cu(CH_3_CN)_4_/(*S*)-Quinox-*t*-BuAd_2_ as the nonracemic catalytic system [143].

**Scheme 79:**
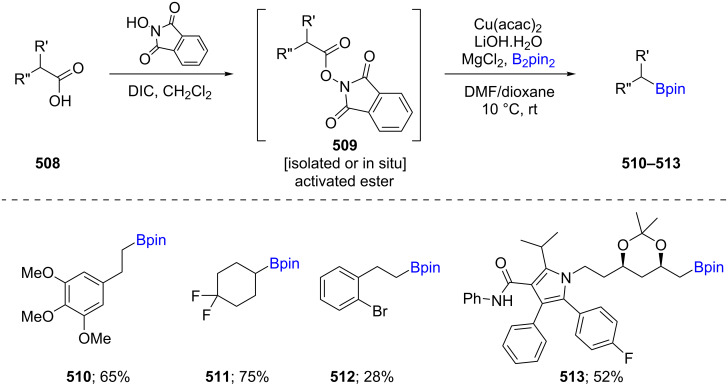
Cu-catalyzed decarboxylative borylation of carboxylic acids.

Baran et al. reported an inexpensive and ligand-free Cu-catalyzed decarboxylative method for the synthesis of boronic esters **510**–**513** through reaction of an acid with *N-*hydroxyphthalimide, via the in situ-generation of a redox active ester **509** (Scheme 79). The reaction conditions reported are mild, the reactions are fast, and they tolerate a wide variety of functional groups leading to the Bpin-containing products in moderate yields. By contrast, substrates bearing halides were low yielding due to unavoidable protodehalogenation or alternative borylation processes [144].

**Scheme 80:**
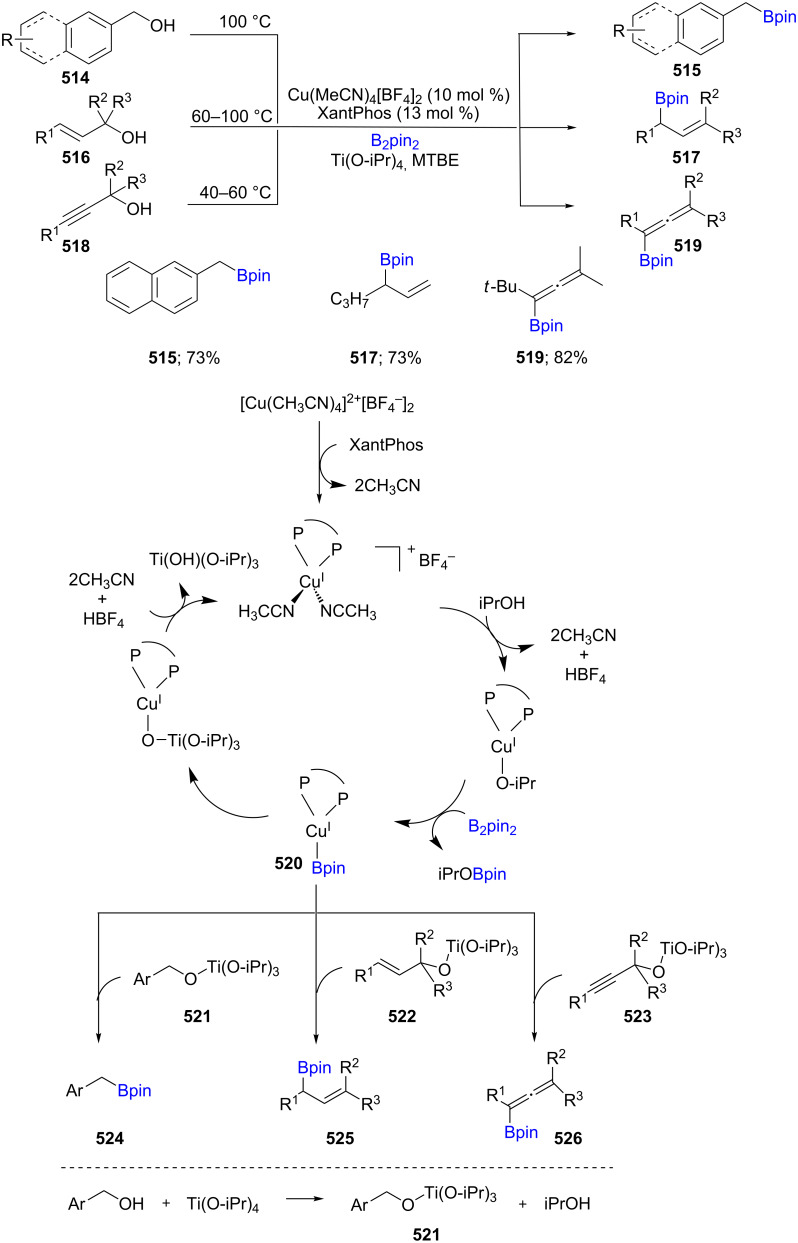
Cu-catalyzed borylation of benzylic, allylic, and propargylic alcohols.

Direct functionalization of sp^3^ C–O bonds present in alcohols is of great interest due to their abundance in nature as well as their commercial availability. As a result, benzylic, allylic, and propargylic alcohols (**514**, **516**, **518**) were employed in the Cu-catalyzed borylation reactions in the presence of B_2_pin_2_ to afford benzyl-, allyl-, and allenyl boronates, respectively (**515**, **517**, **519**). Structural variations in the alcohols did not appear to inhibit reactivity under the reaction conditions. Mechanistic studies suggested a nucleophilic substitution of Cu(I)–Bpin onto an activated hydroxy-Lewis acid adduct. This reaction proceeds under mild conditions, shows a broad substrate scope, and gives the targeted benzyl-, allyl-, and allenyl boronate products in good chemical yields. Ti(O-iPr)_4_ is used in the reaction to activate the alcohol via formation of **521** and iPrOH. The Cu(I) species, formed through reaction of Cu(II) and Xantphos, reacts with iPrOH, followed by B_2_Pin_2_ to generate the intermediate L-Cu-Bpin. The *in situ*-formed L-Cu-Bpin then reacts with species **521**, **522**, and **523** to form the desired borylated products **524**, **525**, or **526**, respectively (Scheme 80) [145].

## Conclusion

Copper-catalyzed processes as described herein provide an excellent access to both carbon–silicon and carbon–boron intermediates. Given the plethora of known chemical methods utilizing such C–Si and C–B bonds en route to derived, valuable functionalities, it is not surprising that considerable attention has been paid to the developments described herein. Moreover, copper has been, and still is, very attractively priced, being classified as a base metal, and remains an incentive, further encouraging future discoveries. Its participation in achiral and, perhaps more noteworthy, asymmetric catalyses, stems in large measure from both its phosphine and *N*-heterocyclic carbene-derived complexes that impart especially useful reactivity and selectivity profiles. And while most of the chemistry discussed to date takes place in organic solvents, its expansion into alternative reaction media (e.g., water) is sure to offer new, exciting, and sustainable opportunities for copper catalysis in the future.
